# Deciphering the Mechanism of Inhibition of SERCA1a by Sarcolipin Using Molecular Simulations

**DOI:** 10.3389/fmolb.2020.606254

**Published:** 2021-02-04

**Authors:** Thomas Barbot, Veronica Beswick, Cédric Montigny, Éric Quiniou, Nadège Jamin, Liliane Mouawad

**Affiliations:** ^1^Institute for Integrative Biology of the Cell (I2BC), CEA, CNRS, Université Paris-Saclay, Gif-sur-Yvette, France; ^2^Physics Department, Evry-Val-d'Essonne University, Paris-Saclay University, Evry, France; ^3^CNRS UMR9187 / INSERM U1196, Institut Curie, PSL Research University, Université Paris-Saclay, Orsay, France

**Keywords:** normal mode analysis, molecular simulations, molecular modeling, calcium ATPase, SERCA1a, sarcolipin, membrane protein

## Abstract

SERCA1a is an ATPase calcium pump that transports Ca^2+^ from the cytoplasm to the sarco/endoplasmic reticulum lumen. Sarcolipin (SLN), a transmembrane peptide, regulates the activity of SERCA1a by decreasing its Ca^2+^ transport rate, but its mechanism of action is still not well-understood. To decipher this mechanism, we have performed normal mode analysis in the all-atom model, with the SERCA1a-SLN complex, or the isolated SERCA1a, embedded in an explicit membrane. The comparison of the results allowed us to provide an explanation at the atomic level for the action of SLN that is in good agreement with experimental observations. In our analyses, the presence of SLN locally perturbs the TM6 transmembrane helix and as a consequence modifies the position of D800, one of the key metal-chelating residues. Additionally, it reduces the flexibility of the gating residues, V304, and E309 in TM4, at the entrance of the Ca^2+^ binding sites, which would decrease the affinity for Ca^2+^. Unexpectedly, SLN has also an effect on the ATP binding site more than 35 Å away, due to the straightening of TM5, a long helix considered as the spine of the protein. The straightening of TM5 modifies the structure of the P-N linker that sits above it, and which comprises the ^351^DKTG^354^ conserved motif, resulting in an increase of the distance between ATP and the phosphorylation site. As a consequence, the turn-over rate could be affected. All this gives SERCA1a the propensity to go toward a Ca^2+^ low-affinity E2-like state in the presence of SLN and toward a Ca^2+^ high-affinity E1-like state in the absence of SLN. In addition to a general mechanism of inhibition of SERCA1a regulatory peptides, this study also provides an insight into the conformational transition between the E2 and E1 states.

## Introduction

**Figure 1:**
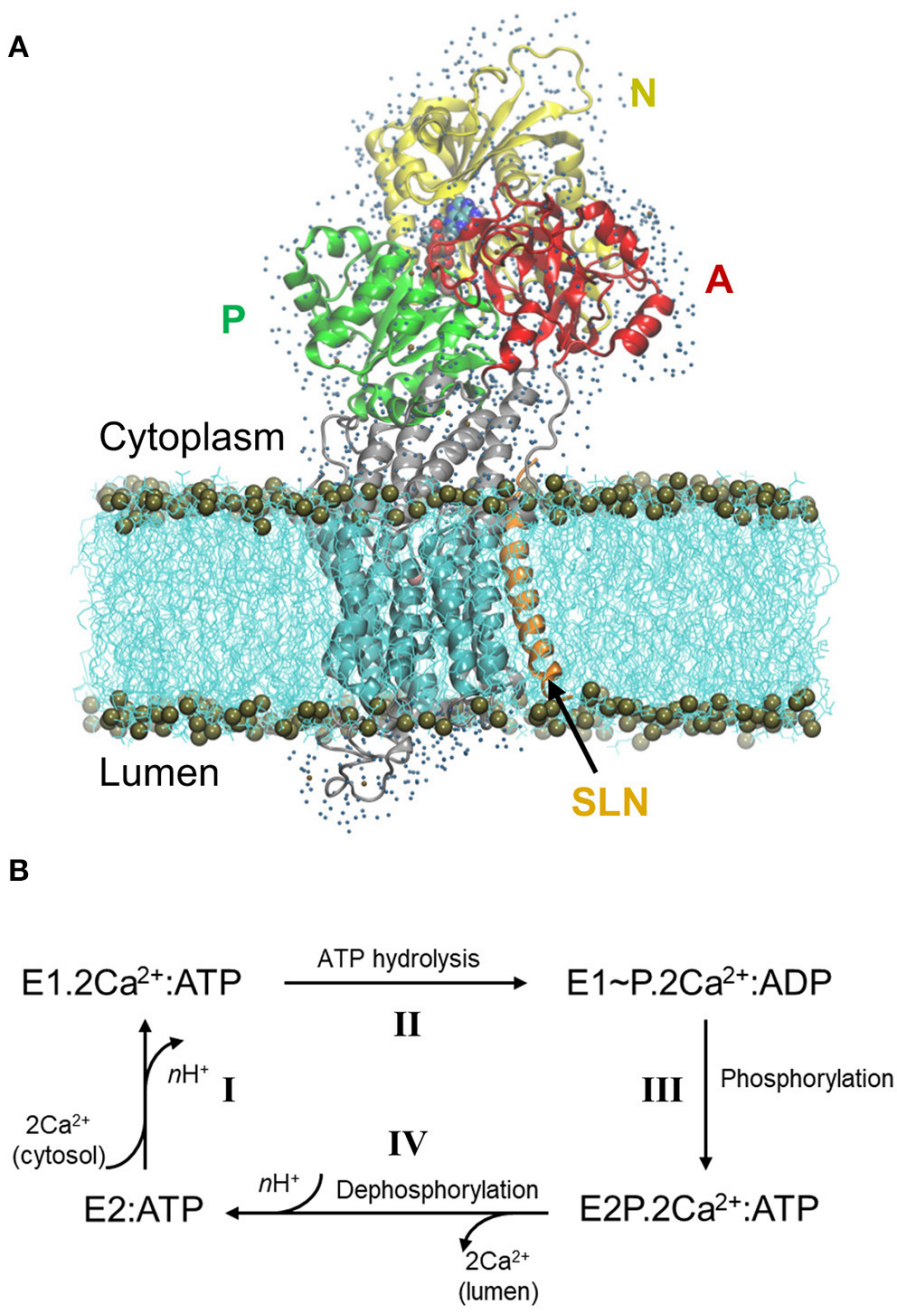
Structure of SERCA1a in the presence of sarcolipin (SLN) and the cycle of Ca^2+^ transport. **(A)** Structure of SERCA1a-SLN embedded in a POPC membrane, with one shell of water surrounding its cytosolic and luminal parts. The N-, P-, and A-domains are in yellow, green, and red, respectively, the TM domain in cyan and the rest of the protein in gray. SLN is in orange and ATP is in spheres colored according to atom name, C: cyan, N: blue, O: red, P: tan, and Mg^2+^: pink. The membrane is in cyan lines with phosphorus atoms in tan spheres. Finally, water molecules are blue dots. **(B)** Simplified cycle of the Ca^2+^ transport from the cytoplasm to the sarcoplasmic reticulum lumen.

The Sarco/Endoplasmic Reticulum Ca^2+^ ATPase (SERCA) is a transmembrane protein that transports Ca^2+^ from the cytoplasm to the sarco/endoplasm, using ATP as an energy source. In the skeletal muscles, SERCA1a, an isoform of the SERCA1 subfamily, is responsible for the muscle relaxation by restocking into the sarcoplasmic reticulum lumen the calcium that was formerly released in the cytosol during muscle contraction. This restocking results in a rapid lowering of the cytosolic Ca^2+^ concentration, which decreases the myosin/actin filaments activity, allowing for muscle relaxation (Hasselbach and Makinose, 1961; Ebashi and Lipmann, 1962). SERCA1a is a 110 kDa protein composed of 10 transmembrane helices (TM1-10) and a large cytoplasmic headpiece that consists of three domains: the Nucleotide-binding (N) domain, which binds an ATP molecule, the Phosphorylation (P) domain, which contains the autophosphorylation site and the Actuator (A) domain, which is responsible for the dephosphorylation of the protein (Figure 1A) (MacLennan et al., 1985; Zhang et al., 1998).

During Ca^2+^ transport across the sarcoplasmic reticulum membrane, SERCA1a undergoes conformational changes from the Ca^2+^ high-affinity state (E1) to the Ca^2+^ low-affinity state (E2). The functional cycle may be described in four main steps (Figure 1B, adapted from Møller et al., 2010): **I-** SERCA1a in the presence of ATP binds two cytoplasmic Ca^2+^ ions to form the **E1.2Ca**^**2+**^**:ATP** complex, in which the two Ca^2+^ binding sites are occluded (PDB ID: 1VFP; Toyoshima and Mizutani, 2004, 3AR2; Toyoshima et al., 2011, 1T5S; Sørensen et al., 2004, and 3TLM; Sacchetto et al., 2012, see Supplementary Table 1 for details about the structures). At this step, two or three protons, H^+^, are released from the calcium-binding sites into the cytoplasm. **II-** Following the occlusion of the Ca^2+^ binding sites, ATP is hydrolyzed to ADP, leading to the formation of a highly energetic intermediate, **E1~P.2Ca**^**2+**^**:ADP** (PDB ID: 2ZBD; Toyoshima et al., 2004 and 1T5T; Sørensen et al., 2004). **III-** The protein is then autophosphorylated and switches to the E2 conformation. The ADP molecule is exchanged with ATP and the Ca^2+^ binding sites open toward the lumen, resulting in the **E2P.2Ca**^**2+**^**:ATP** complex (PDB ID: 5A3R; Clausen et al., 2016 and 3B9B; Olesen et al., 2007). **IV-** The two Ca^2+^ ions are then released into the lumen, accompanied by the protonation of *n* acidic residues (2 ≤ *n* ≤ 3) of the Ca^2+^-binding sites. In this step, auto-dephosphorylation produces the **E2:ATP** complex (PDB code: 3W5C; Toyoshima et al., 2013, only a structure without exogenous molecules like thapsigargin is given), where a structural rearrangement of the transmembrane helices slightly opens the protonated Ca^2+^-binding sites toward the cytoplasm, inducing protons release and enabling the protein to bind cytoplasmic Ca^2+^ again, as in step I. Since an ATP analog is always present in the structures that we consider in this study (except for 3W5C which will be discussed below), from here on, ATP will be omitted from the notation of the states.

The existence of an E1 free-of-Ca^2+^ intermediate state was hypothesized between E2 and E1.2Ca^2+^ states, with the Ca^2+^-binding sites open toward the cytoplasm (Toyoshima et al., 2013; Winther et al., 2013; Espinoza-Fonseca et al., 2014). In this hypothesis, it is unclear if the Ca^2+^-binding sites are free of any ions or if Mg^2+^ or K^+^ ions may replace Ca^2+^. The structure of this intermediate is still unknown, but those of SERCA1a in a putative intermediate E1 state have been proposed in complex with regulatory peptides, like sarcolipin (SLN) (PDB ID: 3W5A; Toyoshima et al., 2013 and 4H1W; Winther et al., 2013) or phospholamban (PLN) (PDB ID: 4KYT; Akin et al., 2013). In these complexes, SERCA1a is in an E1-like state with a large opening of the gate between the calcium-binding sites and the cytoplasm.

The regulatory transmembrane peptide, SLN, composed of 31 residues, is mostly expressed in skeletal muscle cells (Odermatt et al., 1997, 1998). Although it is widely accepted that SLN is an inhibitor of SERCA1a, the detailed mechanism of this inhibition is still not fully understood. The numerous experimental studies of the effect of SLN on SERCA1a clearly show a moderate decrease in Ca^2+^ affinity, whereas contradictory results are obtained concerning the ATP hydrolysis rate, most probably due to the differences in the studied biological systems (Barbot et al., 2016; and references therein).

The two crystal structures of the SERCA1a-SLN complex available in the Protein DataBank (PDB ID: 3W5A; Toyoshima et al., 2013 and 4H1W; Winther et al., 2013) were resolved in the presence of Mg^2+^. One Mg^2+^ is bound in the Ca^2+^ binding sites in 3W5A and two Mg^2+^ in 4H1W. Nonetheless, in both structures, SLN binds to SERCA1a in a similar way, in a groove formed by the transmembrane helices TM2, TM6, and TM9 (Supplementary Figure 1). The two structures are very similar in their transmembrane part, with a root-mean-square deviation (RMSD) over the TM C_α_ atoms of 0.6 Å, whereas the cytoplasmic domains are more open in 3W5A than in 4H1W, yielding to an RMSD over all C_α_ atoms of 2.2 Å.

As described by Toyoshima et al. (2013) and Winther et al. (2013), the most remarkable difference between the crystal structures of SERCA1a (E1.2Ca^2+^, PDB ID: 1VFP), and SERCA1a-SLN (SERCA1a.Mg^2+^:SLN, 3W5A, and SERCA1a.2Mg^2+^:SLN, 4H1W) is the opening of a large mouth in SERCA1a-SLN that leads to the Ca^2+^ binding sites. The opening of this mouth is questioning since, by making these sites more accessible, it is expected to impact the ATPase activity by increasing the Ca^2+^ uptake affinity, while experimental observations show a decrease in the apparent Ca^2+^ affinity. However, this large mouth does not seem to be only due to the presence of SLN, since it is also observed in the structure of SERCA1a.Mg^2+^ in the absence of SLN (3W5B).

In addition to X-ray diffraction experiments, *in silico* experiments were performed to investigate at a molecular level the role of SLN on SERCA1a. This role has been investigated by all-atom molecular dynamics (MD) simulations (Espinoza-Fonseca et al., 2015b; Sahoo et al., 2015; Autry et al., 2016), where analyses were focused on the Ca^2+^ binding sites and the cytosolic part of TM4 (helix M4S4 spanning residues P312-K329). From these analyses, it was concluded that the presence of SLN perturbs the Ca^2+^ binding sites occlusion at E309, leading to an increase in the distance E771-D800, which produces incompetent Ca^2+^ binding sites and allows Ca^2+^ backflux to the cytosol.

Here, we use another *in silico* method, namely Normal Mode Analysis (NMA), to decipher the mechanism of inhibition of SERCA1a by SLN, and to answer the question if this inhibition is due to Ca^2+^ binding, to autophosphorylation, or both. In the NMA method, the dynamics of a protein is approximated by the sum of its internal vibrations. Each normal mode (NM) is a vector corresponding to the direction of the vibration, and the lowest-frequency modes correspond to large-amplitude motions, which are usually equivalent to the concerted motions of the structure. The main interest of NMA is to provide direct access to the principal component motions (the most collective movements), so it is a method of choice to study large conformational changes of proteins. To investigate the effects of sarcolipin on the SERCA1a structure and motion, we chose to study and compare two systems starting from the same PDB structure, SERCA1a.Mg^2+^:SLN (3W5A): one in the presence of SLN, E1.Mg^2+^:SLN, and one from which SLN was removed, E1.Mg^2+^ (see Methods for details). The choice of starting from the same PDB structure, and not from the two crystal structures with and without SLN (3W5A and 3W5B, respectively), was made to enable us to identify the only effects of SLN, without any other considerations, like the difference of the biological material from which SERCA1a was obtained (it was extracted from rabbit muscle for 3W5A and expressed as a recombinant rabbit protein in *Chlorocebus Sabaeus* cells for 3W5B; Toyoshima et al., 2013). However, we have compared a posteriori our results to 3W5B.

Contrary to previous studies on the SERCA1a state-transition, where coarse-grained NMA methods were used—with either only C_α_ atoms (Reuter et al., 2003) or rigid-block residues (Li and Cui, 2002)—, and where the effect of the membrane was not taken into account, here we consider the all-atom model. The protein is embedded in a 1-palmitoyl-2-oleyl-sn-glycero-3-phosphocholine (POPC) bilayer membrane, and its solvent-accessible parts are surrounded by one water shell. To our knowledge, this is the first NMA study of a membrane protein embedded in an explicit membrane, where the entire system is considered as an all-atom model.

## Methods

### Preparation of the Crystal Structure

#### Crystal Structures

The two SERCA1a-SLN structures available in the Protein DataBank (https://www.rcsb.org/), PDB ID: 3W5A (Toyoshima et al., 2013) and 4H1W (Winther et al., 2013), were resolved in the presence of millimolar concentrations of Mg^2+^ instead of Ca^2+^ (40 mM of MgSO_4_ for the former and 75 mM of MgSO_4_ for the latter). They are similar, however, the 3W5A structure has been obtained with a slightly better resolution than 4H1W (3.01 and 3.10 Å, respectively). Besides, in the 4H1W structure, there are some missing residues in the SERCA1a luminal part, i.e., in the L1-2 loop (residues 79 to 86) and in the L8-9 loop (residues 883 to 887). Therefore, we chose to use 3W5A coordinates for this study. In this structure, two chains are available for SERCA1a (chains A and B), but SLN (chain C) is clearly embedded in chain B. Therefore, only chain B (SERCA1a) and chain C (SLN) were considered.

#### Positioning of the Adenosine Triphosphate (ATP)

In the 3W5A crystal structure, in the ATP site, a trinitrophenyl adenosine monophosphate (TNPAMP) is bound, and a Mg^2+^ ion, only chelated by 4 water molecules, is present in its vicinity. To replace TNPAMP with ATP, the crystal structure of SERCA1a in its E1.2Ca^2+^ state was used (PDB ID: 1VFP; Toyoshima and Mizutani, 2004). In this structure, an adenosine-[β, γ-methylene]triphosphate (AMPPCP), an ATP analog, is bound in the ATP pocket. A Mg^2+^ ion is chelated by AMPPCP and 2 water molecules. To do the replacement, in 3W5A, all residues with at least one heavy atom within 5 Å from TNPAMP-Mg^2+^ were selected, and the structure 1VFP was superimposed on these residues. We observed that by this superimposition AMPPCP-Mg^2+^ occupied the same location as TNPAMP-Mg^2+^. Therefore, these coordinates, in addition to those of the two Mg^2+^-chelating waters, were copied to replace those of TNPAMP-Mg^2+^ with their four Mg^2+^-chelating waters. Then AMPPCP was changed into ATP by replacing the β, γ-methylene with an oxygen atom. To adjust the bond lengths between the oxygen atom and the phosphate groups, the energy of the β, γ-phosphates was minimized by 10 steps of the steepest descent method while the rest of the system was kept fixed.

#### Additional Ions and Crystal Water Molecules

In the 3W5A structure, an additional Mg^2+^ ion, chelated by two water molecules, sits in the calcium-binding sites. A Na^+^ ion is chelated by L711, K712, A714, and E732. This monovalent cation is present in almost all SERCA1a structures and plays a stabilizing role in the P-domain Rossman fold (Møller et al., 2010). These additional ions (Mg^2+^ and Na^+^) and the 2 crystal water molecules that chelate the Mg^2+^ ion were also kept in this study.

#### Cleaning the Structure

Since in a crystal structure at a resolution R > 1 Å, the C, N, and O atoms are not distinguishable from each other, all asparagine, glutamine, and histidine residues were carefully examined to define their orientation. Moreover, the pKa of the charged residues were calculated to determine their protonation state. Based on this calculation, no residue of the Ca^2+^ binding site was protonated. All this was done using the protein preparation wizard from the Schrödinger Suite (https://www.schrodinger.com/).

#### Membrane and One Layer of Water

Based on this prepared structure, two systems were considered, one in the presence of SLN, and one where SLN was removed. For both these systems, the transmembrane part of the protein was embedded in a 1-palmitoyl-2-oleyl-sn-glycero-3-phosphocholine (POPC) bilayer using the Orientation of Proteins in Membrane (OPM) database (Lomize et al., 2012) and the Membrane Builder module (Jo et al., 2007, 2009; Wu et al., 2014) from the CHARMM-GUI web server (Jo et al., 2008). Thus, spheres approximating polar lipid heads were randomly created, energy minimized, and equilibrated, and then, the entire lipid atoms were created according to the CHARMM36 parameters. POPC lipids were chosen because: first, phosphatidylcholine (PC) headgroups are the major phospholipid component (70% of the phospholipid content) of the sarcoplasmic reticulum membrane, and second, palmitoyl and oleoyl acyl chains represent 40% of the fatty acyl chains content of this membrane (Gould et al., 1987; Bick et al., 1998). Moreover, POPC lipids assemble in a bilayer with a thickness of about 40 Å in agreement with a functionally optimal membrane thickness for SERCA1a (Starling et al., 1993). The bilayer membrane was made of 376 lipids for SERCA1a-SLN and 379 lipids for SERCA1a alone. The solvation and ionization of the systems were also made using the CHARMM-GUI server. For this, in each system, the two soluble parts of the protein, on the luminal and cytosolic sides, were surrounded by a water box with randomly distributed K^+^ and Cl^−^ ions to reach a concentration of 100 mM. Then, for energy minimizations, only one layer of water and ions around the protein (within 2.8 Å from heavy atoms) was conserved to prevent the artifact shrinkage of the protein due to energy minimizations. Regarding this solvation shell, three systems were considered for the complex SERCA1a-SLN: two in which this water layer was created randomly (they consisted of 1,346 water molecules, 9 K^+^ and 2 Cl^−^ for the first system and of 1,377 water molecules, 7 K^+^ and 2 Cl^−^ for the second system), and one in which this layer was copied as it from the system of the isolated SERCA1a (1,346 water molecules, 6 K^+^ and 4 Cl^−^). Despite the difference of charges and atom numbers, the results were similar. Therefore, in the article only the results with the same water shell around SERCA1a-SLN and SERCA1a (1,346 water molecules, 6 K^+^ and 4 Cl^−^) are presented. In this case, except for the three extra lipids that compensate the absence of SLN in SERCA1a, the only difference between the two systems is the presence or absence of Sarcolipin. All hydrogen atoms were created by the CHARMM-GUI website.

These structures will be energy minimized, as presented below. However, to help us assess their quality after energy minimization, an additional structure of SERCA1a-SLN in the absence of water was created for comparison. The structure was the same as the three other SERCA1a-SLN complexes, considering the presence of SLN, ATP, Mg^2+^, the structural water and Na^+^ ion, and the POPC membrane. The only difference is the absence of the water shell, with its K^+^ and Cl^−^ ions.

### Calculations

#### Potential Energy Force Field

All the following calculations were carried out with program CHARMM (Brooks et al., 1983) using the CHARMM36 all-atom force field (Klauda et al., 2010; Best et al., 2012), excluding the CMAP correction, which is not needed for energy minimizations and normal mode calculations. The TIP3P model (Jorgensen et al., 1983; Neria et al., 1996) was used for water molecules. Electrostatic and van der Waals interactions were switched to zero with a cut-on distance of 6 Å and a cut-off distance of 10 Å, and the relative dielectric constant was equal to 3. These values were chosen to avoid important distortions of the structure due to extreme energy minimizations.

#### Energy Minimizations and Normal Mode Calculations

Each system (protein + membrane + water + ions, and protein + membrane in the absence of water) was energy minimized by 1,000 steps using the steepest descent method followed by conjugate gradient method for thousands of additional steps until the root mean square of the energy gradient (GRMS) fell below 10^−4^ kcal/mol.Å^−1^.

The energy-minimized structures, E1.Mg^2+^:SLN and E1.Mg^2+^, were considered as starting systems to calculate normal modes. Because of the large number of atoms in each system (>70,000 atoms) the DIMB method (Diagonalization in a Mixed Basis) (Mouawad and Perahia, 1993; Perahia and Mouawad, 1995) was used in program CHARMM. DIMB is an iterative method in which the Hessian matrix to be diagonalized is divided into small-size Hessian matrices expressed in a mixed basis of intermediate NMs and Cartesian coordinates. By an iterative process of diagonalizations, the modes converge to those that would be obtained from the diagonalization of the entire Hessian matrix. However, to be able to perform the calculations in a reasonable time (<10 days), we accelerated this method by making it work on a GPU system, in a home-made adaptation of CHARMM. This adaptation was tested beforehand on small proteins to ensure that the results were the same as those obtained on CPU. For each system 206 modes were calculated, the 6 trivial global translation-rotation modes with null frequencies, and 200 non-trivial modes (modes 7 to 206), sorted according to the ascending order of their frequencies. The modes were considered as converged when the maximum eigenvectors convergence was <0.03.

#### Mode overlaps

The percentage of overlap (*p*) between two vectors, ${\vec{V}}_{1}$ and ${\vec{V}}_{2}$ was calculated using their dot product:


$$\begin{array}{l} p={\left[\frac{{\vec{V}}_{1}\cdot {\vec{V}}_{2}}{\left\|{\vec{V}}_{1}\|\cdot \|{\vec{V}}_{2}\right\|}\right]}^{2}\times 100 \end{array}$$

where $\left\|{\vec{V}}_{1}\right\|$ and $\left\|{\vec{V}}_{2}\right\|$ are the norms of vectors ${\vec{V}}_{1}$ and ${\vec{V}}_{2}$.

In this study, we calculated the percentage of overlap (or projection) between the modes of a structure that we call *A* and the difference coordinates vector between structures *A* and *B*. So ${\vec{V}}_{1}={\vec{q}}_{m}^{A}$, where ${\vec{q}}_{m}^{A}$ is mode *m* of structure *A*, with *m*∈[7, 206], and ${\vec{V}}_{2}=\Delta \vec{R}={\vec{R}}^{B}-{\vec{R}}^{A}$, where ${\vec{R}}^{A}$ and ${\vec{R}}^{B}$ are the coordinates of structures *A* and *B*, respectively. Vector $\Delta \vec{R}$ may also be written *A*→*B*. Prior to the projection, structure *B* is superimposed on structure *A* using C_α_ atoms. The projection is also done considering only C_α_ atoms in both vectors, ${\vec{q}}_{m}^{}$ and $\Delta \vec{R}$, in order to avoid misleading directions due to sidechains. The higher the percentage of overlap between these two vectors (${\vec{q}}_{m}$ and $\Delta \vec{R}$), the most straightforward is the transition from *A* to *B*. Here *A* refers to one of the two energy-minimized structures (E1.Mg^2+^ and E1.Mg^2+^:SLN) and *B* to one of the two chosen crystal structures (1VFP for state E1.2Ca^2+^ and 3W5C for state E2). For the E1.2Ca^2+^ state, 1VFP was preferred to the other structures, 3AR2, 1T5S, and 3TLM, despite its slightly lower resolution in some cases, because 1T5S and 3TLM lack the disulfide bridge that stabilizes the protein, and the ATP analog in 3AR2 chelates Ca^2+^ instead of Mg^2+^ (see Supplementary Table 1). For the E2 state, the structure of 3W5C, although lacking an ATP analog, was preferred to 2DQS, because it is free of exogenous molecules like thapsigargin. 2DQS, which has the ATP analog, also contains thapsigargin to stabilize it and disrupt the communication between the transmembrane domain and the cytosolic headpiece of the ATPase (Picard et al., 2006; Montigny et al., 2007). However, despite these differences, the two E2 structures, 3W5C and 2DQS, are very similar, with an RMSD between all their C_α_ atoms of only 0.6 Å.

We also calculated the percentage of overlaps between all the modes calculated from one structure (*B*) over one chosen mode calculated from another structure (*A*), in which case, ${\vec{V}}_{1}={\vec{q}}_{m}^{A}$ and ${\vec{V}}_{2}={\vec{q}}_{n}^{B}$, where *m* = 54 when *A* = E1.Mg^2+^:SLN and *n*∈[7, 206] for *B* = E1.Mg^2+^, or *m* = 40 when *A* = E1.Mg^2+^ and *n*∈[7, 206] for *B* = E1.Mg^2+^:SLN (see subsection The P-Domain Plays a Central Role in the Transition Toward the E2 State in the Results).

#### Atomic Fluctuations

For each structure, E1.Mg^2+^:SLN and E1.Mg^2+^, the atomic fluctuations, *f*_*i*_, were calculated from the 200 modes using:


$$\begin{array}{l} f_{i}=\sqrt{\langle \Delta {\vec{r}}_{i}^{2}\rangle =}\sqrt{k_{B}T\overset{l}{\underset{m=7}{\sum }}\frac{{\vec{q}}_{im}^{2}}{\omega_{m}^{2}}} \end{array}$$

where ${\vec{r}}_{i}$ is the position of atom *i*, ${\vec{q}}_{im}^{}$ its component in mode *m*, ω_*m*_ the frequency of mode *m, T* is the temperature, and *k*_*B*_ the Boltzmann constant. The sum runs from 7 to *l*, which is usually equal to 3*N*, where *N* is the number of atoms. However, the fluctuations are dominated by those of the lowest frequency modes, since $f_{i}^{2}$ is inversely proportional to the frequency. Here *l* = 206. But, even when all modes are considered, the fluctuations obtained from NM in the all-atom model are undervalued compared to those obtained from molecular dynamics or the crystal B-factors. Therefore, for this calculation, *T* was taken equal to 1,300 K to reach the range of the fluctuations obtained from the B-factors.

The fluctuations were also calculated from the crystal structure B-factors using the equation:


$$\begin{array}{l} f_{i}=\frac{1}{\pi }\sqrt{\frac{3}{8}B_{i}} \end{array}$$

#### Correlations

The correlation C_ij_ between two C_α_ atoms, *i* and *j*, calculated from one mode, *m*, is as follows:


$$\begin{array}{l} C_{ij}=\frac{\langle \Delta {\vec{r}}_{i}\Delta {\vec{r}}_{j}\rangle }{\sqrt{\langle \Delta {\vec{r}}_{i}^{2}\rangle \langle \Delta {\vec{r}}_{j}^{2}\rangle }}=\frac{{\vec{q}}_{im}\cdot {\vec{q}}_{jm}}{\sqrt{{\vec{q}}_{im}^{2}{\vec{q}}_{jm}^{2}}} \end{array}$$

#### Analyses and Visualization

All analyses were performed with CHARMM. The plots were drawn using Kaleidagraph (http://www.synergy.com) and the images were done using VMD (Visual Molecular Dynamics, https://www.ks.uiuc.edu/Research/vmd; Humphrey et al., 1996).

## Results

The two energy-minimized structures were named according to their state, E1.Mg^2+^:SLN and E1.Mg^2+^, to avoid confusion with the crystal structures in presence of Mg^2+^, namely SERCA1a.Mg^2+^ (3W5B), SERCA1a.Mg^2+^:SLN (3W5A), and SERCA1a.2Mg^2+^:SLN (4H1W). For both structures, E1.Mg^2+^:SLN and E1.Mg^2+^, the transmembrane part of the protein was embedded in a lipid bilayer made of POPC and the cytoplasmic domains were surrounded by one layer of water with some ions randomly distributed (see Methods for details and Figure 1A). For E1.Mg^2+^:SLN three systems were considered with different water shells and ions. To assess the quality of these structures, and also of E1.Mg^2+^, we followed two separate procedures. First, we used the Swissmodel website (https://swissmodel.expasy.org/assess), where the results were obtained from MolProbity version 4.4 (Chen et al., 2010). In Supplementary Table 2, it can be observed that the geometrical features of E1.Mg^2+^:SLN and E1.Mg^2+^ are improved compared to the starting crystal structure, 3W5A. Second, we calculated the RMSD of the C_α_ atoms of all the energy-minimized structures with respect to both 3W5A and the crystal structure in the absence of SLN, 3W5B. But since absolute numbers do not mean much, we also compared the results with those of a fourth E1.Mg^2+^:SLN structure that we created only for this comparison, and which is expected to be of bad quality, because without any water shell, although the protein is embedded in the membrane. We name it E1.Mg^2+^:SLN(nw), for no water. As observed in Supplementary Figure 2A, the RMSD of the structures in the presence of water is around 1 Å, when compared to 3W5A or 3W5B, and it reaches 3 Å for E1.Mg^2+^:SLN(nw). This shows that, in the presence of water, there was no significant deformation of the structure, which is not the case in the absence of water. As expected, in the absence of water the protein shrank a little, and its radius of gyration diminished by about 1.5 Å (Supplementary Figure 2B). So, as planned, the structure E1.Mg^2+^:SLN(nw) was completely discarded from this study. For the three E1.Mg^2+^:SLN structures in the presence of water, the obtained NM results were qualitatively similar. Therefore, only one E1.Mg^2+^:SLN system is presented here: the one with exactly the same shell as E1.Mg^2+^.

Since it has been proposed that the crystal structure of the SERCA1a-SLN complex is in an intermediate state between the Ca^2+^ low-affinity state (E2) and the Ca^2+^ high-affinity state (E1.2Ca^2+^), the role of SLN in the conformational transition of SERCA1a toward E2 or E1.2Ca^2+^ was first investigated. For this comparison, two PDB structures were chosen based on their properties given in Supplementary Table 1 (see Methods section for details), 1VFP (chain A) for E1.2Ca^2+^, and 3W5C for E2.

### E1.Mg^2+^:SLN Has the Propensity to Go Toward E2 and E1.Mg^2+^ Toward E1.2Ca^2+^

**Figure 2:**
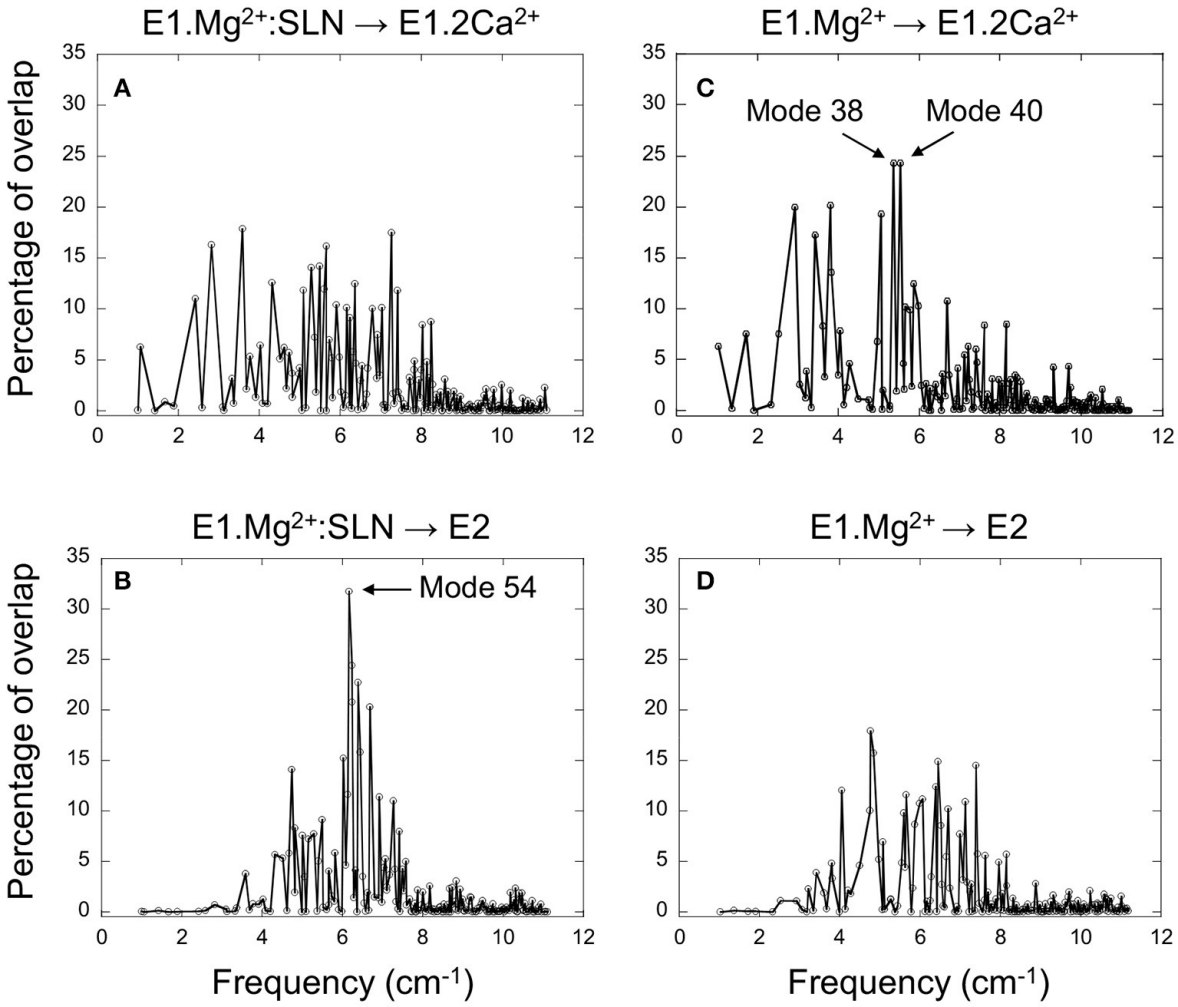
Projection of the normal modes on the difference coordinate vectors. Percentage of overlaps between the 200 NMs of E1.Mg^2+^:SLN and the difference vectors E1.Mg^2+^:SLN → E1.2Ca^2+^ **(A)** and E1.Mg^2+^:SLN → E2 **(B)**. Percentage of overlaps between the 200 NMs of E1.Mg^2+^ and the difference vectors E1.Mg^2+^ → E1.2Ca^2+^ **(C)** and E1.Mg^2+^ → E2 **(D)**. E1.Mg^2+^:SLN and E1.Mg^2+^ are energy-minimized structures and E1.2Ca^2+^ and E2 are crystal structures (PDB ID: 1VFP and 3W5C, respectively). Only C_α_ atoms are considered for these projections.

The 200 lowest-frequency modes of each energy-minimized system, E1.Mg^2+^:SLN (${\vec{q}}_{m}^{\text{E1}.\text{M}{\text{g}}^{\text{2}+}:\text{SLN}}$) and E1.Mg^2+^ (${\vec{q}}_{m}^{\text{E1}.\text{M}{\text{g}}^{\text{2}+}}$), were projected on the difference coordinate vector with either E1.2Ca^2+^ (PDB ID: 1VFP; $\Delta \vec{R}={\vec{R}}^{\text{E1}.\text{M}{\text{g}}^{\text{2}+}:\text{SLN}}-{\vec{R}}^{\text{E1}.\text{2C}{\text{a}}^{\text{2}+}}$ or $\Delta \vec{R}={\vec{R}}^{\text{E1}.\text{M}{\text{g}}^{\text{2}+}}-{\vec{R}}^{\text{E1}.\text{2C}{\text{a}}^{\text{2}+}}$) or E2 (PDB ID: 3W5C; $\Delta \vec{R}={\vec{R}}^{\text{E1}.\text{M}{\text{g}}^{\text{2}+}:\text{SLN}}-{\vec{R}}^{\text{E2}}$ or $\Delta \vec{R}={\vec{R}}^{\text{E1}.\text{M}{\text{g}}^{\text{2}+}}-{\vec{R}}^{\text{E2}}$). The results are presented in Figure 2, where the percentages of overlaps are plotted vs. the NM frequencies. We observe that the lowest-frequency modes, which usually correspond to the internal most collective motions of a protein, do not present high overlaps with the difference vectors. This is because these modes are only collective when considering the entire system, i.e., the protein plus the membrane and the water shell. Here these modes mainly correspond to the global vibrations of the rectangular membrane, in which the transmembrane domain is embedded. Therefore, the TM domain follows these vibrations, in addition to a global motion of the three cytosolic domains with respect to the membrane. When we consider the modes of slightly higher frequency, global internal motions of the protein itself are observed, resulting in higher overlaps.

For the E1.Mg^2+^:SLN structure, when its modes are projected on vector E1.Mg^2+^:SLN → E2 the highest overlap is 32%, obtained for mode 54, the frequency of which is 6.16 cm^−1^, whereas when projected on vector E1.Mg^2+^:SLN → E1.2Ca^2+^, the highest overlap is only 18% (Figures 2A,B). This shows the preference of E1.Mg^2+^:SLN to go toward E2 than toward E1.2Ca^2+^.

Conversely, in the absence of SLN, for E1.Mg^2+^, the highest percentage of overlap, 24%, is observed for two modes corresponding to motions toward E1.2Ca^2+^, modes number 38 and 40, with frequencies 5.38 and 5.54 cm^−1^, respectively (the modes are ranked according to the ascending order of their frequencies), whereas, toward E2, the highest overlap was only 18% (Figures 2C,D). This shows a higher tendency of E1.Mg^2+^ to go toward E1.2Ca^2+^ than toward E2.

To decipher the role of the different domains of SERCA1a in the conformational changes required for the transition toward the E1.2Ca^2+^ or E2 states, the three modes that gave the highest percentage of overlap (mode 54 of E1.Mg^2+^:SLN and modes 38 and 40 of E1.Mg^2+^) were further analyzed.

### The P-Domain Plays a Central Role in the Transition Toward the E2 State

**Figure 3:**
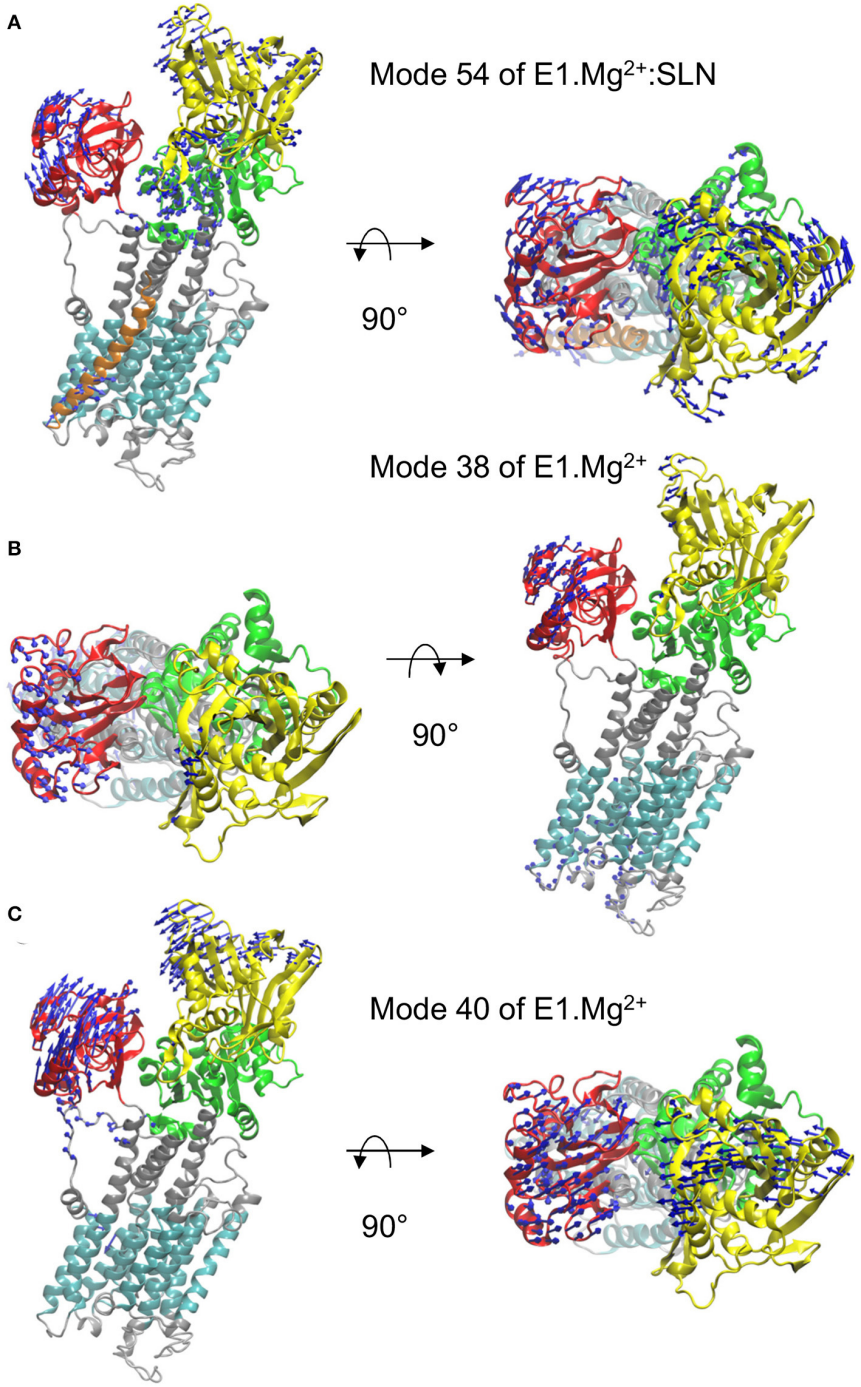
Motions along the three modes identified from the overlaps. In each of the three panels, a profile view and a top view of the protein are presented. The same color code as in Figure 1A is used, i.e., the N-, P-, and A-domains are in yellow, green, and red, respectively, the TM domain in cyan and the rest of the protein in gray. The directions of motions are shown as blue arrows. The lengths of the arrows are proportional to the amplitude of displacement of the C_α_ atoms when the entire system is displaced by mass-weighted RMSD of 3 Å. For clarity, the arrows corresponding to displacement amplitudes under 3 Å are omitted. The motions are shown along mode 54 of E1.Mg^2+^:SLN **(A)** and along modes 38 **(B)** and 40 **(C)** of E1.Mg^2+^.

The motion of E1.Mg^2+^:SLN along mode 54, which brings the structure toward E2, is shown in Figure 3A. It corresponds to the rotation of the A-domain around the region of the P-domain which is in contact with it. The P-domain rotates in the opposite direction around the TM5 helix, and the N-domain, which is in the continuity of the P-domain, rotates in the same direction. The TM domain does not present any wide motions, except for the N-terminus of helix TM2, which is in contact with the C-terminus of SLN and moves in concert with it.

**Figure 4:**
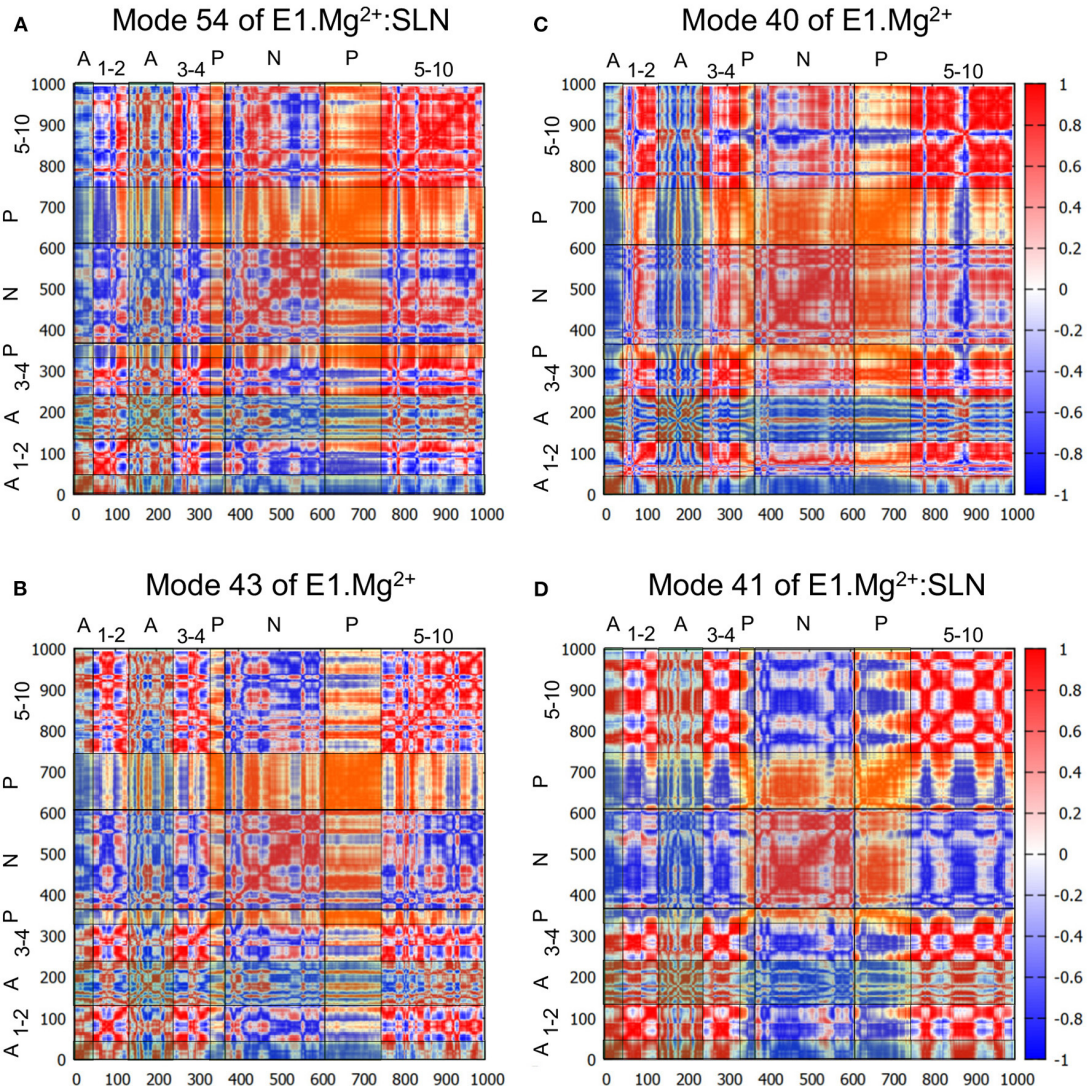
Correlation heatmaps. The correlations are calculated from modes 54 **(A)** and 41 **(D)** of E1.Mg^2+^:SLN and modes 43 **(B)** and 40 **(C)** of E1.Mg^2+^. The red color corresponds to high correlations, white to the absence of correlations, and blue to high anticorrelations. However, to facilitate the delimitation of the cytosolic domains, transparent vertical and horizontal strips were overlaid on each heatmap, with the following color code: N-domain: gray, P-domain: yellow, and A-domain: cyan, which modifies the perception of the correlation colors. The names of the domains are added on the top and the left of each heatmap, where the numbers refer to the TM helices.

To further describe the concerted motions of the different domains of SERCA1a for mode 54, the correlations from mode 54 of E1.Mg^2+^:SLN were calculated (Figure 4A). The heatmap of these correlations shows two compact regions, the TM5 to TM10 sub-domain and the P-domain, that move in a concerted way (although with different amplitudes as observed in Figure 3A). Whereas, the TM5-TM10 sub-domain is mainly correlated with the P-domain, the P-domain is also correlated with the N-domain and anticorrelated (concerted in the opposite direction) with TM1, TM2, and the A-domain. TM1 and TM2 are two of the three helices that link the A-domain to the rest of the protein.

Does a similar motion exist in the absence of SLN, i.e., in E1.Mg^2+^, that was not detected with the projections on the difference coordinates vector? To answer this question, we projected all the calculated modes of E1.Mg^2+^ on mode 54 of E1.Mg^2+^:SLN. The highest percentage of overlap is 60% for mode 43 of E1.Mg^2+^ (frequency = 5.65 cm^−1^). However, this mode presents only a percentage of overlap of 12% with the difference vector E1.Mg^2+^ → E2. The analysis of this mode shows similar motions of the protein as those along mode 54 of E1.Mg^2+^:SLN, but with a smaller amplitude and involving less extended regions of the protein (Supplementary Figure 3A). The heatmap of the correlations calculated from mode 43 of E1.Mg^2+^ (Figure 4B) shows that the P-domain is still the only one that presents global correlations with the rest of the protein. However, the correlation coefficients are smaller than those calculated from mode 54 of E1.Mg^2+^:SLN, due to slight differences in the directions of motion.

In conclusion, in the transition toward E2, first, the P-domain is the only compact region that presents global correlations, whether they be positive or negative, with the rest of the protein; and second, while in the presence of SLN, these correlations are strong, coinciding with a good propensity for going toward E2, in the absence of SLN these correlations are decreased as well as the propensity for the E2 transition. So we conclude that the P-domain plays a central role in the transition toward E2.

### A Close Correlation Between the P- and N-Domains Is Important for the Transition Toward the E1.2Ca^2+^ State

In the absence of SLN, i.e., for structure E1.Mg^2+^, two modes have been identified for the transition toward E1.2Ca^2+^, modes 38 and 40 (Figure 2C). The motion along mode 38 is not collective and is mainly located in long flexible loops (mostly in the lumen and a few in the A and N domains) (Figure 3B). Due to this location, the vibration along this mode cannot drive the state transition, and thus for the study of the state transition this mode is not further considered. The motion along mode 40 is collective and resembles a hinge-bending motion that brings the A- and N-domains close to each other, like in the E1.2Ca^2+^ state. In addition, it brings the kinked helix TM1—and more specifically residue L60, the junction between the two parts of TM1—near TM4, starting to close the protein mouth. These data highlight the concerted motion between the cytoplasmic domains and the occlusion of the Ca^2+^ binding sites, as previously suggested from structural analyses (Sørensen et al., 2004; Toyoshima and Mizutani, 2004).

The heatmap of the correlations calculated from mode 40 (Figure 4C) shows that in this case, the P- and N-domains act together as a concerted subunit, which is anticorrelated with the A-domain. This result is consistent with the hinge-bending-like motion observed for this mode (Figure 3C).

To investigate whether a mode of E1.Mg^2+^:SLN with a motion similar to that of mode 40 of E1.Mg^2+^ exists, all modes of E1.Mg^2+^:SLN were projected on mode 40 of E1.Mg^2+^. The best percentage of overlap (48%) was obtained for mode 41 (frequency 5.50 cm^−1^) of E1.Mg^2+^:SLN. Although this mode has almost the same frequency as mode 40 of E1.Mg^2+^, the motions of the cytoplasmic domains along it are of smaller amplitudes and they are more localized (Supplementary Figure 3B). Its moderately low frequency is probably due to a relatively large amplitude motion of the TM domain along the normal to the membrane plane. This motion of the TM domain is not observed in the transition toward the E1.2Ca^2+^ state. The correlation heatmap corroborates this observation, where the P- and N-domains are less correlated with each other, but highly anticorrelated with all the rest of the protein (Figure 4D). Therefore, the presence of SLN seems to hamper the transition toward E1.2Ca^2+^, because at the same time it decreases the correlation between the P- and N-domains and it correlates the closing hinge-bending motion with an upward movement of the TM domain, which is not necessary for this transition.

### SLN Reduces the Flexibility of the Ca^2+^ Gating Residues

The fluctuations of the C_α_ atoms of SERCA1a were obtained from the 200 modes that were calculated for each system. These fluctuations show that, for both systems, the cytoplasmic A-, N-, and P-domains of the protein fluctuate more than its transmembrane domain (Supplementary Figure 4), reflecting the degree of accessibility to solvent of each region and therefore, its freedom of movement. We observe that these results are different from the fluctuations obtained from the thermal B-factors of the crystal structure of SERCA1a.Mg^2+^:SLN (PDB ID: 3W5A), where they are reversed; in the PDB structure, the TM domain is more fluctuating than the cytoplasmic domains due to the crystal packing. Indeed, in the crystal unit cell, the cytoplasmic domains are in close contact with other proteins of the cell, which is not the case of the TM domain. Therefore, the experimental fluctuations (B-factors) profile does not reflect the flexibility of SERCA1a.Mg^2+^:SLN in a membrane.

**Figure 5:**
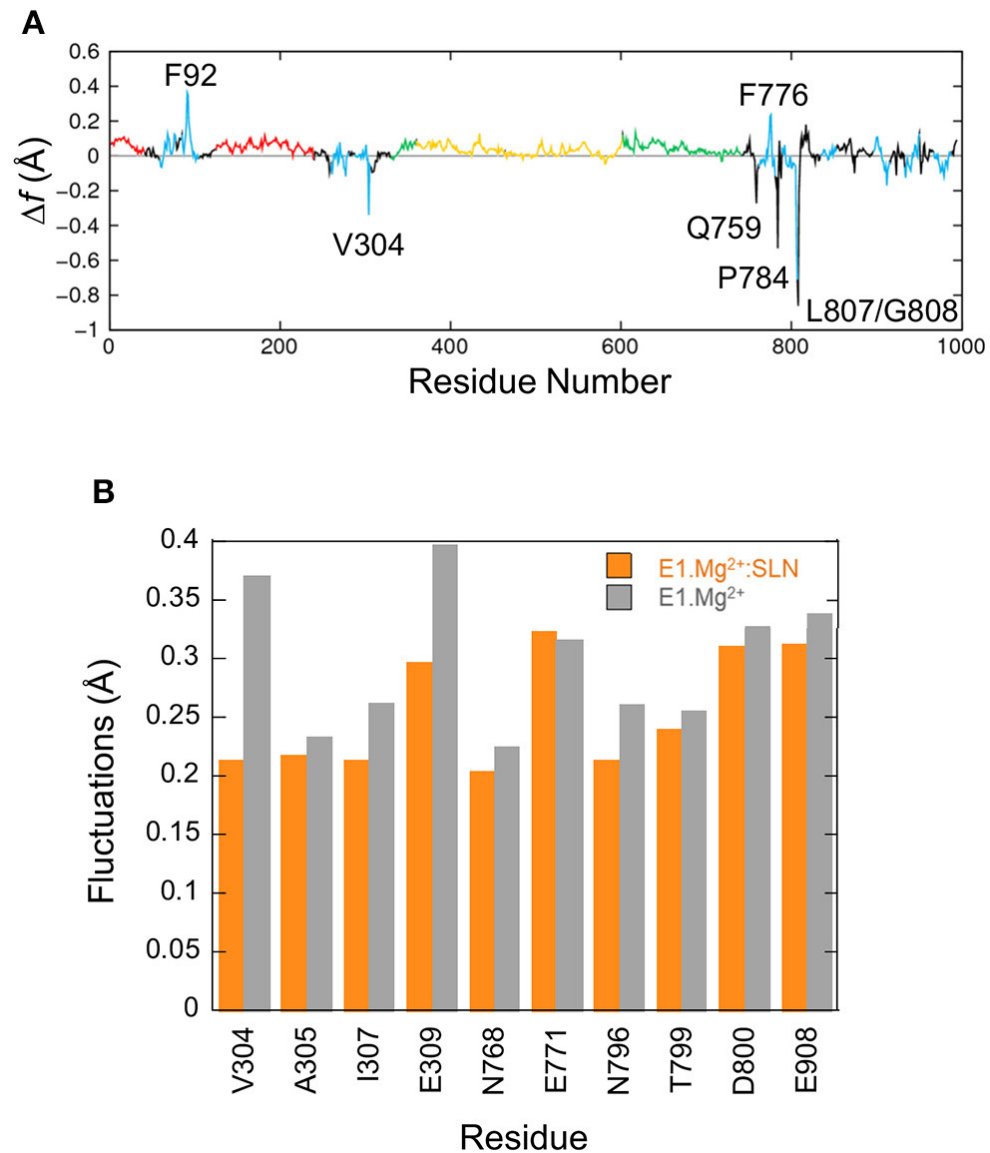
Fluctuations of residues. **(A)** Δ*f* , the difference of fluctuations of the C_α_ atoms calculated from the 200 modes. The fluctuations of E1.Mg^2+^ were subtracted from those of E1.Mg^2+^:SLN. The same color code as in Figure 1A is used to delimit the domains. **(B)** Average fluctuations of the Ca^2+^ chelating groups, i.e., the carboxyl, hydroxyl or carbonyl groups of the residues in the two Ca^2+^ binding sites. Orange bars for E1.Mg^2+^:SLN and gray bars for E1.Mg^2+^.

The fluctuation profiles in the presence and absence of SLN are very similar, so to observe the difference between them the curve of E1.Mg^2+^ was subtracted from that of E1.Mg^2+^:SLN. The difference curve (Figure 5A) shows that the observable modifications (>|0.2| Å) are very localized. For four residues (V304, Q759, L807, and G808) these modifications are observed in the difference between E1.Mg^2+^ and all three E1.Mg^2+^:SLN structures for which the calculations were done (Supplementary Figure 5). So, they are robust and reliable. However, around the three other residues (F92, F776, and P784), the modifications seem rather fortuitous, because they are only observed in the difference with the E1.Mg^2+^:SLN structure that is presented here. Moreover, these three residues have no obvious link with the state-transition mechanism that emerges from all our results, and their mutations were not described to experimentally modify the active transport of Ca^2+^ in contrast to data concerning V304, Q759, L807, and G808. This shows the robustness of NM method that allows us to discriminate between meaningful and fortuitous fluctuations. Considering V304 in TM4, Q759 in TM5, and L807-G808 in TM6, the fluctuations decrease in the presence of SLN. Q759, L807, and G808 are in close contact with each other, and the reason for the decrease in their fluctuations will be given below in subsection Due to the Straightening of TM6 and TM5. The most interesting residue is V304, because it is part of the Ca^2+^ binding site II, and represents, with E309, its opening gate. This needs a more thorough analysis, which is given below. It should be noted that all the binding site fluctuations are similar in the three E1.Mg^2+^:SLN structures.

**Figure 6:**
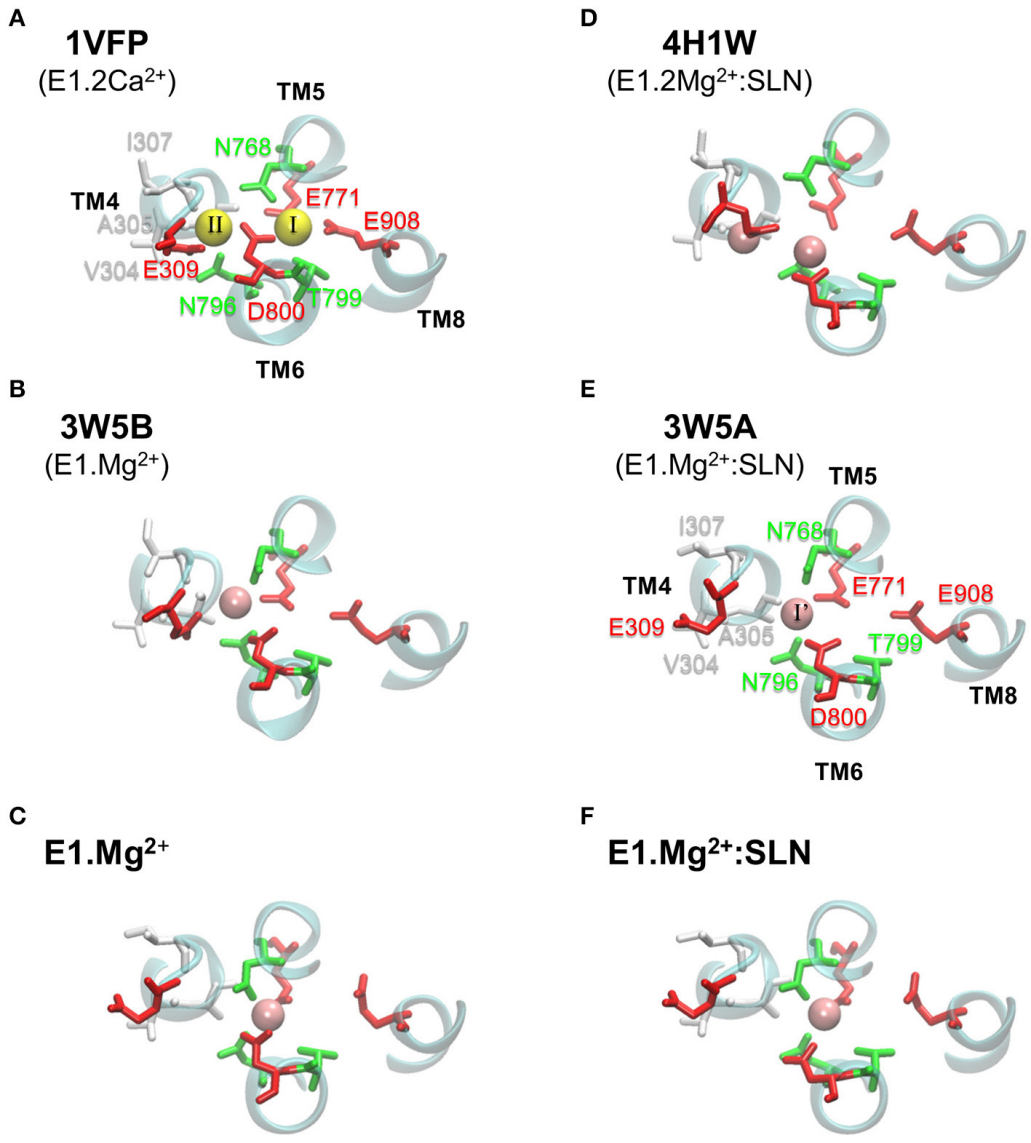
Ca^2+^ and Mg^2+^ binding sites in various SERCA1a structures. In **(A–C)** the structures are without SLN, and in **(D–F)** they are in the presence of SLN. **(A**,**B**,**D,E)** show PDB structures, with the PDB ID written in bold and the corresponding state given below the ID. **(C,F)** present the two energy-minimized structures, in the absence and presence of SLN, respectively. In each panel, all the residues of the two Ca^2+^ binding sites are drawn, even when they are not chelating any ion, as for the structures with Mg^2+^. Ca^2+^ and Mg^2+^ ions are yellow and pink spheres, respectively. The binding site residues are colored according to their type, acidic: red, polar: green, and hydrophobic: white. **(A)** In site I, Ca^2+^ is chelated by eight oxygen atoms, of which four are from the carboxyl groups of acidic residues (TM5:E771, TM6:D800, and two from TM8:E908), two from the sidechains of polar residues (TM5:N768 and TM6:T799), in addition to two buried water molecules. In site II, Ca^2+^ is chelated by seven oxygen atoms, of which only three are from the carboxyl groups of acidic residues (two from TM4:E309 and one from TM6:D800), one is from the sidechain of a polar residue (TM6:N796) and three are from the carbonyl groups of the backbone of hydrophobic residues (TM4:V304, A305 and I307). **(E)** In 3W5A, Mg^2+^ sits in site I', located between sites I and II and it is exclusively chelated by residues from these two sites, in addition to two water molecules. For clarity, water molecules are omitted and only small parts of helices TM4, TM5, TM6, and TM8 are drawn as cyan cartoons. Also for clarity, we chose to annotate only two structures, one with Ca^2+^ (1VFP) and one with Mg^2+^ (3W5A).

In its E1 state, SERCA1a can bind two Ca^2+^ ions in two adjacent sites, I and II, located between four transmembrane helices, TM4, TM5, TM6, and TM8, as observed in the structure of the E1.2Ca^2+^ state, 1VFP (Figure 6A). Although Ca^2+^ ions enter from site II, they bind sequentially, first to site I then to site II, as shown by the studies using ^45^Ca^2+^ isotopes (Orlowski and Champeil, 1991a,b), and the ion is more tightly chelated in site I due to its higher electronegativity (see the legend of Figure 6).

In the crystal structures with Mg^2+^ ions (3W5A, 3W5B, and 4H1W), the metal sits in an intermediate site, named I', located between sites I and II, and encompassing a part of their residues (Figures 6B,D,E). Site I' is of a smaller volume than sites I and II, which makes it more appropriate to accommodate Mg^2+^ as mentioned by the authors of the two structures with SLN (Toyoshima et al., 2013; Winther et al., 2013).

Here we focus on the chemical groups of all residues in sites I and II that chelate Ca^2+^, because they include all residues of site I' that chelate Mg^2+^. So we calculated for E1.Mg^2+^:SLN and E1.Mg^2+^ the average fluctuations of the carboxyl groups of E309, E771, D800, and E908, the hydroxyl group of T799, the sidechains carbonyl groups of N768 and N796, and the backbone carbonyl groups of V304, A305, and I307. The results are presented in Figure 5B. Only the fluctuations of the two gating residues, V304 and E309, clearly decrease in the presence of SLN. This difference of flexibility suggests that the presence of the peptide may hamper the access of the cation to its binding sites. This idea is supported by experimental observations where mutation of V304 by a more voluminous residue, V304L, slightly diminishes the Ca^2+^ binding affinity, although V304 only chelates Ca^2+^ by its backbone carbonyl (Sørensen et al., 2004). In addition, the identified mutations of the other gating residue, E309Q, E309D, E309A, E309K, E309L, E309M, and E309F, generally prevent chelation of Ca^2+^ in site II and therefore significantly decrease the Ca^2+^ affinity (Clarke et al., 1989; Andersen and Vilsen, 1992; Vilsen and Andersen, 1992; Falson et al., 1997; Menguy et al., 1998, 2002; Sorensen and Andersen, 2000; Zhang et al., 2000; Inesi et al., 2002; Lenoir et al., 2006; Clausen et al., 2013). V304 is located at the C-terminus of the membrane part of helix TM4, and E309 in the hinge region (^308^PEG^310^) just before the N-terminus of the cytosolic part of TM4, also named helix M4S4. Due to the break of TM4, its cytosolic part (M4S4) is bent with respect to the plane of the membrane. In the presence of SLN, salt bridges are established between R324 and K328 at the C-terminus of M4S4 on the one hand, and SLN E2 on the other hand. Energy minimizations did not modify the bending angle of this helix, even in the absence of SLN. However, the salt bridges observed in the presence of SLN exerted a strain on M4S4. So V304 and E309, which are in the bending region on the opposite side of this helix, are probably hampered in their motion. In the absence of SLN, helix M4S4 is released, increasing the fluctuations of these two residues.

### SLN Weakens the Chelation of Ca^2+^ at Site I

Observation of the structures of the Ca^2+^ binding sites in the presence and absence of SLN after energy minimizations shows that they are similar except for residue D800 (Figures 6C,F). D800 is a key residue for calcium binding as, in the presence of Ca^2+^, it points toward both sites I and II to chelate the two calcium ions. In the crystal structure of SERCA1a.Mg^2+^:SLN (3W5A), which is the basis of this study, D800 is a little far for the chelation of Mg^2+^, the distance between the ion and the closest atom of D800 being 2.87 Å (Figure 6E). Toyoshima et al., 2004 suggested that this could be the consequence of the approximate positioning of Mg^2+^ due to the low resolution (around 3 Å) of the crystal structure. But in fact, D800 is far from site I, not only from Mg^2+^. This may be represented by the distance between the two attractive groups COO^−^ of D800 and NH_2_ of N768. N768 was chosen because it has the only sidechain with an attractive interaction with D800, while it is not located at the same helix (D800 is at TM6 and N768 at its facing helix TM5). More precisely, the distance was calculated between any oxygen atom, O_δ*_, of the D800 carboxylate group and atom N_δ2_ of the N768 amide group. This distance is 5.65 Å in the crystal structure of SERCA1a.Mg^2+^:SLN (3W5A), which is much bigger than that of E1.2Ca^2+^ (1VFP), where it is 3.08 Å (Supplementary Table 3). Besides, D800 does not point toward site II either. Nonetheless, in the presence of SLN, the relaxation of the structure by energy minimizations did not correct sufficiently this effect, since D800 did not point well toward site I, although the O_δ*_-N_δ2_ distance decreases to 4.83 Å, which is still more than 50% larger than in the presence of Ca^2+^ (PDB ID: 1VFP). Conversely, energy minimizations in the absence of SLN brought the O_δ*_-N_δ2_ distance to 3.40 Å, which is comparable to E1.2Ca^2+^ (3.08 Å).

**Figure 7:**
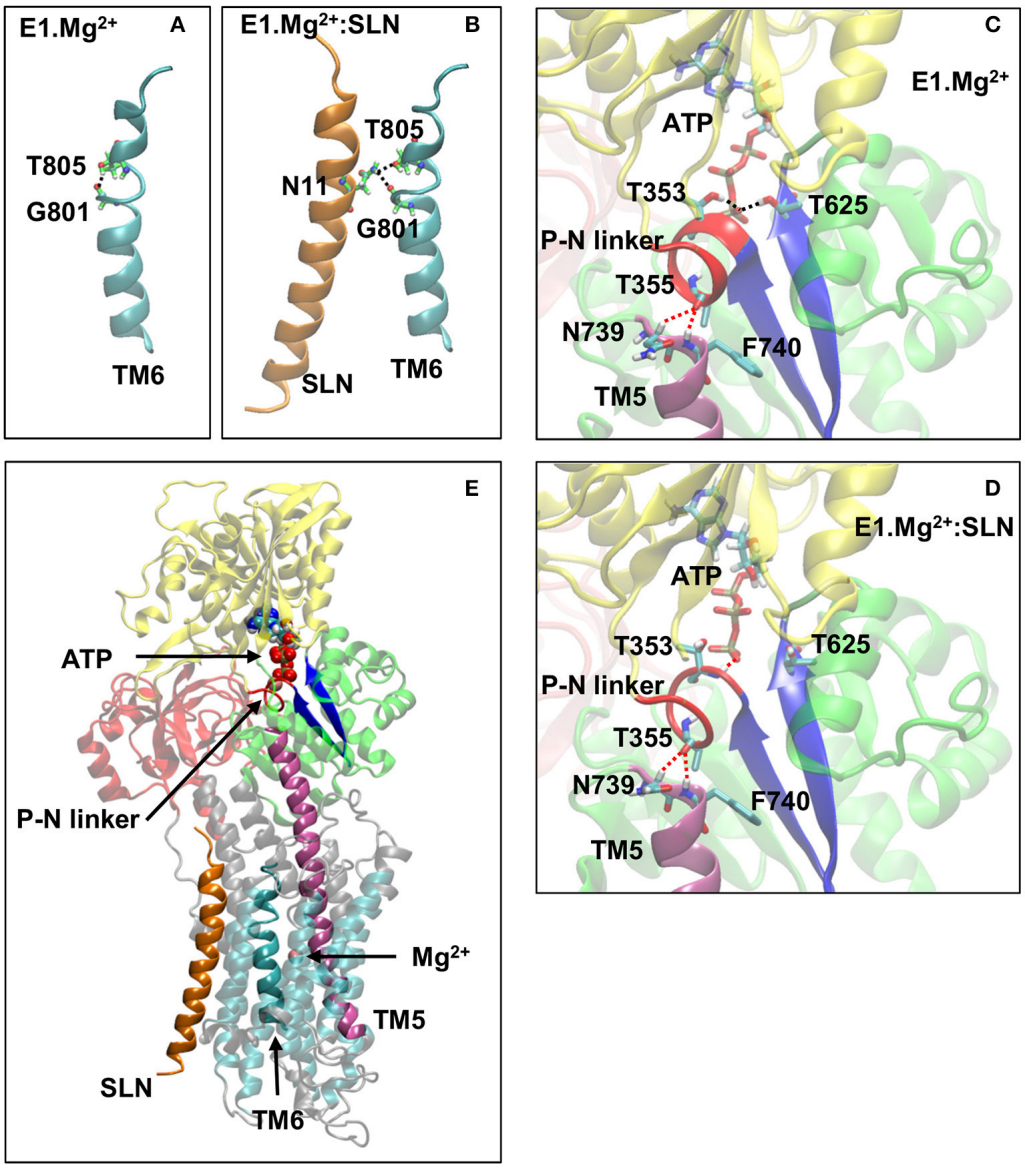
A few key structural modifications between E1.Mg^2+^ and E1.Mg^2+^:SLN. **(A)** and **(B)** Modification of the TM6 H-bonds in the absence and presence of SLN, respectively. **(C)** and **(D)** Modifications in the vicinity of ATP and the P-N linker in the absence and presence of SLN, respectively. D351 and Mg^2+^ of the ATP are not shown for clarity. The important H-bonds are in black dashed lines and some distances in red dashed lines. In both cases, the distances between the hydrogen and oxygen atoms that are related by these dashed lines are as follows: **(C)** in E1.Mg^2+^, T355-N739 = 3.3 Å, T355-N740 = 2.4 Å, ATP-T353 = 1.9 Å, and ATP-T625 = 2.0 Å and **(D)** in E1.Mg^2+^:SLN, T355-N739 = 2.5 Å, T355-N740 = 2.5 Å, ATP-T353 = 2.2 Å, and ATP-T625 = 5.6 Å. **(E)** Zoom out where the protein is shown in transparency except for the elements that play an important role in the transmission of information from SLN to ATP to highlight their location. These elements are SLN, TM5, TM6, the P-N linker, the two β-strands that hold T625 and D351, ATP, and the Mg^2+^ ion in the Ca^2+^ binding site. Generally, the same color code as in Figure 1A is used for the domains and the explicit atoms. However, in **(A,B)** carbon atoms are in green to be distinguished from TM6, and in **(C–E)**, the P-N linker is in red, TM5 is in purple and the two β-strands that hold T625 and D351 are in dark blue.

This distinct positioning of D800 may be due to the different structuration of helix TM6, to which belongs D800. Indeed, in the absence of SLN, TM6 kinks at 2/3 of its length, in the region ^800^DGLP^803^, because of the presence of a glycine and a proline, as reported for most of the structures of SERCA1a in the absence of SLN (Soulié et al., 1999; Toyoshima, 2009; Møller et al., 2010). The N-terminal part of TM6 (residue 789 to 800) is structured as an α-helix, whereas its C-terminal part (residues 801 to 810) comprises a 3–10 helix turn (Figure 7A). The α- and 3–10 helices are separated by an unstructured link, which lacks the intra-helix *i, i*+4 hydrogen bond between C=O of G801 and N-H of T805. This bond is replaced with a hydrogen bond between the carbonyl group of G801 and the hydroxyl group of the T805 sidechain. In the presence of SLN, the latter H-bond is broken and both C=O of G801 and OH of T805 sidechain establish hydrogen bonds with the facing N11 sidechain of SLN (Figure 7B). These new H-bonds result in a slight straightening of helix TM6 with a little rotation around its axis. Due to this constraint, residue D800, which neighbors G801 and T805, is not free anymore to point toward the Ca^2+^ binding site I. In the presence of Ca^2+^ instead of Mg^2+^, we assume that this constraint impedes D800 from adopting the right position for the chelation of one of the two calcium ions in the presence of SLN, resulting in a decrease of affinity for Ca^2+^. This result is in line with the findings of Espinoza-Fonseca et al. (2015a) and Fernández-de Gortari and Espinoza-Fonseca (2018) where MD simulations were carried out on SERCA1a in the presence of phospholamban (PLN), a regulatory peptide similar to SLN. The H-bonds between residue N34 of PLN (the equivalent of N11 in SLN) and G801 and T805 of SERCA1a were shown to make the Ca^2+^ binding sites incompetent.

### The Presence of SLN Affects the Phosphorylation Site …

In addition to the Ca^2+^ binding sites, which are in its vicinity, SLN seems to also affect the phosphorylation site, which is distant by more than 35 Å. In the crystal structures, the ATP analog, when present, is positioned in the N-domain, with its phosphate groups pointing toward ^351^DKTG^354^, a motif common to all P-type ATPases, which comprises D351, the residue to be phosphorylated (Møller et al., 2010; and references therein). The autophosphorylation of the protein consists of the formation of an aspartyl-phosphate through a nucleophilic association between the ATP γ-phosphate and the carboxyl group of D351 (Ridder and Dijkstra, 1999). To achieve the phosphorylation process, residues T353 and T625 (both in the P-domain) were described to interact through their side chains with the γ-phosphate to stabilize it. These two threonine residues are critical for proper orientation of the ATP γ-phosphate group preceding autophosphorylation (Maruyama et al., 1989; Clausen et al., 2001; Ma et al., 2003, 2005). In the E1.2Ca^2+^ crystal structure (1VFP), the γ-phosphate of the ATP analog, adenosine-[βγ-methylene]triphosphate (AMPPCP), establishes hydrogen bonds with these two residues, T353 and T625, whereas, in the crystal structure of SERCA1a.Mg^2+^:SLN (3W5A), the γ-phosphate of the ATP analog, trinitrophenyl adenosine monophosphate (TNPAMP), is far from them. This difference could have been attributed to the nature of the ATP analogs in the two structures (1VFP and 3W5A) since TNPAMP is not as similar to ATP as AMPPCP. However, in both our structures, where TNPAMP was replaced with ATP (see Methods for details), this different position of ATP is still observed after energy minimizations. Indeed, the ATP γ-phosphate stays far from T353 and T625 in the presence of SLN, whereas in the absence of SLN, it comes close and establishes hydrogen bonds with the hydroxyl groups of the two threonines, as in the E1.2Ca^2+^ crystal structure. This shows that the distance between the ATP γ-phosphate and the two threonines is influenced by the presence of SLN.

Analyses of both energy-minimized structures, with and without SLN, showed that the region around T353 is unstructured in the presence of SLN. This region consists of residues 352 to 357, dubbed here the P-N linker as it links the P- and N-domains (Figures 7C–E). It almost encompasses the entire autophosphorylation ^351^DKTG^354^ motif. In the absence of SLN, the P-N linker adopts a π-helix structure, which brings the N-H group of T625 and both N-H and O-H groups of T353 to establish hydrogen bonds with the ATP γ-phosphate. Therefore, starting from the same initial structure, 3W5A, energy minimizations in the absence of SLN brought the protein to a conformation that stabilizes the ATP γ-phosphate in an intermediate position that precedes autophosphorylation, whereas, this was not the case in the presence of SLN. So the question is how does SLN affect the ATP position, which is more than 35 Å away.

### …Due to the Straightening of TM6 and TM5

In both energy-minimized structures, E1.Mg^2+^:SLN and E1.Mg^2+^, the P-N linker is in close contact with F740, which is the N-terminus of helix TM5 (Figures 7C–E). The N-terminal half of this long helix (residues 740 to 759) is cytoplasmic, whereas its C-terminal half (residues 760 to 780) is embedded in the membrane and is in contact with TM6 (residues 789 to 810). The curvature of the cytoplasmic half of TM5 changes (Supplementary Figure 6); it straightens in the presence of SLN due to the relative straightening of the 3–10 helix of TM6, which takes place because of the hydrogen bond of G801 and T805 of this helix with N11 of SLN, as described above in subsection SLN Weakens the Chelation of Ca^2+^ at site I. Indeed, the straightening of TM6 results in the reorientation of the neighboring L807, which comes closer to Q759 of TM5 and pushes it, promoting TM5 straightening. The produced steric hindrance explains the decrease of the fluctuation of residues Q759, L807, and G808 in the presence of SLN as presented above in subsection SLN Reduces the Flexibility of the Ca^2+^ Gating Residues and Figure 5A. Mutations of these residues show their importance for SERCA1a activity. Indeed, the mutant Q759A presents a lower turn-over rate (74%) than the wild type (WT), which is accompanied by phosphorylation slowdown (t_1/2_ is 0.26 s^−1^ for WT and 0.43 s^−1^ for Q759A) (Sorensen and Andersen, 2000). Additionally, mutant L807A has a significantly reduced affinity for calcium (Ca_1/2_ drops from 0.35 μM for WT to about 5 μM for mutant) but with a moderate effect on the calcium transport rate. Mutant G808A is much slower (only 50% of the calcium transport rate of the WT), although with only a marginal effect on calcium binding (Ca_1/2_ of about 0.3 μM for the mutant; Rice and MacLennan, 1996).

The difference of curvature of TM5 has an effect on the relative position of the P-N linker: in the presence of SLN, the C=O group of T355, which is located in the middle of the linker, is equidistant from the two N-terminal residues of TM5, N739, and F740 (the distance of the carbonyl O of T355 from the C_α_ aliphatic hydrogen of N739, O–H_α_ = 2.5 Å, and from the hydrogen of the amide group of F740 O-HN = 2.5 Å), whereas in its absence, this carbonyl group stays almost at the same distance from F740 (distance O-HN = 2.4 Å) but substantially farther from N739 (distance O–H_α_ = 3.3 Å). Consequently, in the presence of SLN, the P-N linker is more constrained, as it is stuck on the top of TM5 equidistantly from N739 and F740. Site-directed mutagenesis has demonstrated that mutations of all the residues of the P-N linker, except G354A, result in a slow enzyme phosphorylation turnover, showing the importance of this linker in the phosphorylation process (Maruyama et al., 1989). The N-terminal part of TM5 has also a crucial role in mediating communication between the calcium-binding sites and the catalytic domain (Sorensen and Andersen, 2000), which was shown even before resolving the first crystal structures (Andersen, 1995). Replacement of F740 with leucine, a residue almost as bulky as phenylalanine, moderately affects the enzyme turnover rate (83% of the WT), whereas this rate drops down to <10% when F740 is replaced with alanine (Sorensen and Andersen, 2000).

## Discussion

### The Metal-Binding Sites

Comparison of the various crystal structures of the E1.2Ca^2+^ state, like 1VFP and 3AR2, shows that the Ca^2+^ binding sites I and II are well defined, with similar positions of the two Ca^2+^ ions and their chelating residues (for 1VFP, see Figure 6A). This is not the case for the crystal structures obtained in presence of Mg^2+^, whether SLN is present or not. Indeed, the Mg^2+^ binding site I', is not well-defined because there are doubts about the position of Mg^2+^ and its chelating residues (Figures 6B,D,E), due to a poor resolution in that region, as mentioned by the authors (Toyoshima et al., 2013). In the crystal structures 3W5A (SERCA1a.Mg^2+^:SLN) and 3W5B (SERCA1a.Mg^2+^ in the absence of SLN), the cation is placed near N768 and far from D800, whereas in 4H1W (SERCA1a.2Mg^2+^:SLN), the cation in site I' is placed near D800 and far from N768. In the latter structure, a second cation is trapped at the entrance of the binding sites between E309 and V304, “stabilizing an open structure” (Winther et al., 2013). Considering the two energy-minimized structures generated here from 3W5A, in the absence of SLN the Mg^2+^ ion is chelated by both N768 and D800, whereas in its presence, D800 cannot come close enough to chelate Mg^2+^ because of the hydrogen bonds between the neighboring G801, T805 on the one hand and SLN-N11 on the other hand (Figure 7B). This was also observed in MD simulations in the presence of either SLN (Espinoza-Fonseca et al., 2015b) or PLN (Espinoza-Fonseca et al., 2015a). We assume that this would also be the case in the presence of Ca^2+^, or maybe, since Ca^2+^ is bigger than Mg^2+^, D800 would possibly be able to chelate only one Ca^2+^ but not two as it does in the absence of SLN. This would, in any case, slightly reduce the affinity for calcium in the presence of SLN, which is in good agreement with the effect of sarcolipin on the Ca_1/2_ that drops from 0.35 ± 0.02 to 0.51 ± 0.02 μM as described earlier (Odermatt et al., 1997). Remarkably, the mutation of SLN-N11 to alanine restores almost completely the calcium affinity with a Ca_1/2_ 0.39 ± 0.03 μM. Additionally, by its proximity to G801 and T805, which directly interact with SLN, and also its proximity to TM5, D800 seems to play a role, not only in the binding of the cation but also in the transmission of information from the Ca^2+^ binding sites to the phosphorylation site. The observation of the experimental effects of mutations D800N and D800E, in the absence of SLN, shows that both the calcium transport and the phosphorylation turnover are 2-fold slower (Clarke et al., 1989; Andersen and Vilsen, 1992; Vilsen and Andersen, 1992; Zhang et al., 2000; Inesi et al., 2002).

At the entrance of the Ca^2+^ binding sites, the distance between the C_α_ atoms of V304 and E309 in E1.Mg^2+^ is slightly smaller than in E1.Mg^2+^:SLN. Such a difference is also measured between these C_α_ distances within the PDB structures, 3W5A and 3W5B, with and without SLN, respectively. However, these two residues are substantially more flexible in the absence of SLN, probably due to the absence of strain exerted by the peptide on the C-terminus of helix M4S4. This flexibility is necessary, at least for E309, to chelate the cation. The lower flexibility of these residues in the presence of SLN also results in a slight reduction of the Ca^2+^ affinity as recently reported by Montigny et al. (2021).

### The Phosphorylation Site

The two energy-minimized structures E1.Mg^2+^:SLN and E1.Mg^2+^ are similar, with an RMSD over all C_α_ atoms around 1 Å. Nevertheless, some important local differences were highlighted in the results, notably in the P-N linker, the TM5 and TM6 helices, and the position of ATP. Indeed, in the presence of SLN, the P-N linker is unstructured as in the E2-state crystal structures (3W5C, 2DQS) and TM5 and TM6 helices are straightened (Supplementary Figure 6A). ATP is in a relatively high, deep position into the N-domain—with a distance between the top ATP atom (N1) and its closest residue M494 (atom S) of 3.93 Å—, which does not allow it to establish H-bonds with T353 and T625 (for the distances between the ATP γ-phosphate and these residues, refer to the legend of Figures 7C,D). In the absence of SLN, the P-N linker adopts a π-helix structure, helix TM5 is slightly more curved and TM6 slightly more kinked than in the presence of SLN. Therefore, ATP can come in a lower and shallower position relative to the N-domain (the distance ATP:N1–M494:S = 5.15 Å), allowing it to establish H-bonds with both T353 and T625.

These differences are also observed, at least qualitatively, between the two crystal structures SERCA1a.Mg^2+^:SLN (3W5A) and SERCA1a.Mg^2+^ (3W5B), although these structures are overall similar (RMSD ≈ 1 Å). Indeed, in 3W5B (SERCA1a.Mg^2+^), the P-N linker is structured, helix TM5 slightly more curved, and TM6 slightly more kinked than in 3W5A (SERCA1a.Mg^2+^:SLN) (Supplementary Figure 6B). However, TNPAMP, the ATP analog in these crystal structures, is not close to T353 and T625 in 3W5B (SERCA1a.Mg^2+^), like in the E1.Mg^2+^ energy-minimized structure. This is probably due to strong electrostatic interactions (or H-bonds although they are not observed as such) between the nitro groups of TNPAMP (which are not present in ATP) and surrounding arginines and lysines (K515, R560, and R678) of the N-domain. Therefore, in the crystal structure 3W5B (SERCA1a.Mg^2+^), the position of TNPAMP is not relevant for the protein activity. On the other hand, the energy-minimized structure E1.Mg^2+^ was compared to 1VFP and 3AR2, the two crystal structures of E1.2Ca^2+^, comprising the SERCA1a disulfide bridge and an ATP analog, AMPPCP, more similar to ATP. The closer position of ATP to T353 and T625 residues, observed in E1.Mg^2+^, was also observed in 1VFP and 3AR2. In the latter structures, the γ-phosphate establishes H-bonds with the hydroxyl groups of both T353 and T625 as in E1.Mg^2+^. Remarkably, in these structures, the P-N linker adopts a π-helix structure accompanied by the curvature of TM5 and the kink of TM6, as in 3W5B (SERCA1a.Mg^2+^) and our energy-minimized E1.Mg^2+^ structure. These local modifications around the phosphorylation site may slightly slow down the ATP hydrolysis rate as observed in Odermatt et al. (1998), Asahi et al. (2002), Tupling et al. (2002), Hughes and Middleton (2003), MacLennan et al. (2003), Douglas et al. (2005), Buffy et al. (2006), Hughes et al. (2007), Gorski et al. (2013), and Barbot et al. (2016).

Several experimental studies (Odermatt et al., 1998; Douglas et al., 2005; Buffy et al., 2006) showed that SLN slightly reduces the ATPase activity at low calcium concentration (lower than its *K*_*d*_), whereas at high Ca^2+^ concentration, the ATPase activity is restored. Indeed, since the work of Lee's group in 2002 (Smith et al., 2002), it has been suggested that, at high Ca^2+^ concentration, SLN may also induce uncoupling or slippage of SERCA1a, i.e., the phosphorylation rate remains constant, whereas calcium is not transported anymore. Our results on the phosphorylation site were obtained in the presence of only one metal ion, therefore, they are comparable with the experimental results at low calcium concentration. They are in good agreement with the observations that SLN produces a decrease of the hydrolysis rate. However, our results don't allow us to exclude that, in other experimental conditions, like higher calcium concentrations, an uncoupling may be observed.

### Comparison of the Effects of Sarcolipin and Phospholamban on SERCA1a

The structure of SERCA1a in the presence of PLN, another regulatory peptide, is also known (Akin et al., 2013). PLN is mainly expressed in cardiac and smooth muscle cells (Kirchberber et al., 1975). It is a peptide of 52 residues composed of a cytosolic N-terminal part of 23 residues and a transmembrane C-terminal part of 29 residues. The transmembrane part of PLN binds to SERCA1a in a way similar to SLN (Akin et al., 2013) and it has similar effects on the protein activity (MacLennan and Kranias, 2003), although the sequence alignment of the TM part of PLN and SLN shows that only two adjacent residues are fully conserved, namely N11 and F12 of rabbit SLN (see also Supplementary Figure 2 in Montigny et al., 2021). The alignment was done with the SEAVIEW program (Galtier et al., 1996; Guindon et al., 2010) using all non-redundant sequences from various organisms given in UniProt (https://www.uniprot.org), i.e., 108 organisms for PLN and 110 for SLN. Interestingly, the conserved residue N11 contributes to the straightening of SERCA1a TM6 by establishing H-bonds with SERCA1a-G801 and -T805, which are also fully conserved in 116 non-redundant sequences of SERCA1a. Comparison of the structure of SERCA1a-PLN (4KYT) with those of SERCA1a-SLN (3W5A and 4H1W) shows that they are similar, despite the absence of cations in the Ca^2+^-binding sites in the structure with PLN. However, SERCA1a-PLN is closer to 3W5A (RMSD = 1.6 Å on all C_α_ atoms) than to 4H1W (RMSD = 2.6 Å). In the structure of SERCA1a-PLN (4KYT), the P-N linker is unstructured and helices TM5 and TM6 straightened like in all the crystal structures of SERCA1a-SLN (3W5A, 4H1W) and the energy-minimized structure E1.Mg^2+^:SLN. This observation suggests that SLN and PLN follow a similar mechanism for the inhibition of SERCA1a, especially that this inhibition is lost when SERCA1a-T805 is mutated to alanine (Rice and MacLennan, 1996; Asahi et al., 1999, 2003).

However, there are some differences between the two peptides apart from the additional N-terminal domain of PLN. For instance, the luminal C-terminal tail of SLN consists of a highly conserved sequence, ^27^RSYQY^31^. This sequence is not present in PLN, whereas it was shown that, in SLN, it contributes to the activity of the peptide, and more precisely, R27 and Y31 were found to be essential for SLN function (Gorski et al., 2013). When a chimera of PLN was created by adding this tail to its C-terminus, the PLN activity was enhanced to reach that of the wild-type SLN. The authors attributed the importance of R27 and Y31 to their π-π and cation-π interactions with aromatic residues of SERCA1a like F88 and F92. But these residues are too far for such interactions; SLN R27 and Y31 point in the opposite direction from SERCA1a (Supplementary Figure 7). In this study, we observed that the region of SERCA1a around F92, which is in contact with Y29 of the SLN C-terminal tail, fluctuates with this tail. But since this enhanced fluctuation was not confirmed in all three E1-Mg^2+^:SLN energy-minimized structures, we considered it as fortuitous. The probable role of SLN R27 and Y31 is to anchor SLN to the membrane by interacting with the charged PC headgroups of the POPC lipids. Thus, these interactions could stabilize the SERCA1a-SLN complex in the membrane and therefore, increase the SLN inhibitory function.

While PLN is not associated with SERCA1a all along the cycle (Chen, 2015), SLN seems to remain bound to the ATPase throughout the cycle of the pump (Sahoo et al., 2013, 2015). So, despite the incompetence of the Ca^2+^ binding sites described above, the metal seems to be able to bind to the Ca^2+^ sites, even in the presence of SLN. There is most likely an equilibrium between the interaction of residue SERCA1a-D800 with the cation on the one hand, and the interaction of SERCA1a-G801 and -T805 with SLN-N11 on the other hand. So, we hypothesize that, for high Ca^2+^ concentration, the equilibrium should be displaced toward the interaction between D800 and the cation. This does not necessarily imply the dissociation of SLN from SERCA1a. Interestingly, the presence of such an equilibrium between SERCA1a and PLN was demonstrated by MD simulations (Fernández-de Gortari and Espinoza-Fonseca, 2018), where the equilibrium displacement did not provoke the dissociation of the peptide.

### Deciphering the Propensity for the Transition Toward E2

The results presented above revealed that, in the presence of SLN, the motions of SERCA1a toward the E2 state are possible, whereas those toward the E1.2Ca^2+^ state are penalized, although E1.Mg^2+^:SLN is already in an E1-like state. Conversely, in the absence of SLN, although the structure is similar to that in the presence of SLN, the motions toward E2 are penalized, whereas the transition toward the E1.2Ca^2+^ state is now more favorable. This tends to confirm that the E1.Mg^2+^:SLN state is an intermediate conformation between E2 and E1.2Ca^2+^, as proposed by Toyoshima et al. (2013) and Winther et al. (2013), and that SLN impedes the motions that bring the structure close to E1.2Ca^2+^. Analyses of the motions along the modes and of the structural differences between E1.Mg^2+^:SLN and E1.Mg^2+^ provide us with an explanation for these observations. Indeed, to go toward E2, the P-domain undertakes a rotation around helix TM5 and entails the rotation of the N-domain in the same direction and of the A-domain in the opposite direction, like a cogwheel. In the presence of SLN, the peptide pulls the 3–10 helix part of TM6 a little away from TM5 (except for L807 whose sidechain then straightens and comes closer), leaving enough room for TM5 to straighten up and to adapt to the P-domain rotation, which makes this rotation, and therefore the transition toward E2, comfortable. In the absence of SLN, TM6 leans on TM5, modifying the curvature of the helix and increasing its interactions with the P-domain, which makes the independent rotation of the domain, and therefore the transition toward E2, more difficult.

### Deciphering the Propensity for the Transition Toward E1.2Ca^2+^

For the transition toward E1.2Ca^2+^, the N-domain undertakes, with the A-domain, a closing movement, similar to the hinge-bending motion. This movement may be described as a rotation of the N-domain around the axis of the P-N linker. In the presence of SLN, concomitantly to this rotation, TM5 undertakes an upward movement, whereas this helix is rather straight, with almost all its expected *i, i*+4 backbone H-bonds present. So TM5 behaves as a rigid body along its axis, and during its upward movement, it squeezes the unstructured P-N linker, which sits on the top of it, pushing it toward the N-domain. This movement presents an important stress and results in an anticorrelation between the N-domain and TM5, as observed in the heatmap of mode 41 of E1.Mg^2+^:SLN (Figure 4D). Also, because ATP is positioned a little deeper into the N-domain, compared to its position in the absence of SLN, the N-domain encounters more difficulty during its rotation movement. All this together impedes the transition toward E1.2Ca^2+^ in the presence of SLN.

In the absence of the peptide, ATP is in a shallower position, releasing the N-domain rotation. In addition, the P-N linker is structured and slightly moved away from the top of TM5, which allows a little sliding of TM5 around the linker. TM5 does not undertake an upward movement similar to that in the presence of SLN, hence the absence of its anticorrelations with the N-domain (Figure 4C), but its sliding around the linker is accompanied by a slight compression of the helix along its axis. However, since TM5 is rather curved, with a little number of its *i, i*+4 backbone H-bonds disrupted, it is less rigid and its slight compression is softened, which makes the transition of the protein toward E1.2Ca^2+^ possible in the absence of SLN.

### The Central Role of the P-Domain

The transition toward either E2 or E1.2Ca^2+^ proved to be governed by either the motion of the P-domain or around the P-N linker, which is usually considered as part of the P-domain. These motions are determined by the conformation of helix TM5, which, in its turn, is affected by the structural modifications of TM6 due to the presence of SLN. The importance of the P-domain in the state transition of SERCA1a was hypothesized by Møller et al. (2010) based on structural analyses of the protein, in addition to the conservation of this domain. Indeed, the P-domain is the most highly conserved part of SERCA, and this is true for P-type ATPases in general (Møller et al., 1996).

## Conclusion

Based on Normal Mode calculations and Energy Minimizations, we may summarize the mechanism of action of SLN on SERCA1a as follows. When SLN binds to SERCA1a, it establishes hydrogen bonds between its N11 residue on the one hand and SERCA1a T805 and G801 on the other hand, which induces a pulling of the 3–10 helix part of TM6 toward SLN and away from TM5. This movement has effects on both the Ca^2+^ binding sites and the phosphorylation site. For the former, a restraint is then introduced on D800, which is located in TM6, preventing it from chelating the metal. Concomitantly, the two gating residues at the entrance of the binding sites, V304 and E309, become less flexible, making the entrance of the metal more difficult. This decreases the affinity of SERCA1a for Ca^2+^, although the structure is in an E1-like state. The second effect is on the phosphorylation site, more than 35 Å away from SLN. This effect is mediated by helix TM5, which straightens when TM6 is pulled away from it by SLN. The straightening up of TM5 destructures the P-N linker that sits above it, hampering ATP from coming close to D351 (the residue to phosphorylate), and therefore decreasing the phosphorylation rate. Besides, the presence of SLN hinders the transition toward the E1.2Ca^2+^ state because of the position of ATP, deeper in the N-domain, and of the destructuration of the P-N linker, which impedes the sliding of the N-domain around this linker for a hinge-bending-like motion. Conversely, the transition toward E2 is facilitated by the presence of SLN, because, by moving TM6 a little away from TM5, SLN contributes to reducing the steric hindrance on TM5, enabling it to adapt to the P-domain rotation, necessary for this transition. Finally, this mechanism can be generalized to other inhibitory peptides, such as PLN, that bind to SERCA1a in a manner similar to that of SLN.

## Data Availability Statement

The datasets presented in this article are not readily available because a part of the data will be used for another article. Requests to access the datasets should be directed to Liliane Mouawad, liliane.mouawad@curie.fr. And the author detected the following words and expressions: Global list: Protein DataBank, UniProt.

## Author Contributions

TB did the calculations and analyzed the results under the supervision of LM. EQ adapted the method DIMB for GPU and tested it on small proteins. VB, CM, and NJ provided the subject, compared the simulation results to experimental results, and participated in the manuscript writing. LM wrote the first draft of the article. LM and VB coordinated the editing of the manuscript. All authors contributed to the article and approved the submitted version.

## Conflict of Interest

The authors declare that the research was conducted in the absence of any commercial or financial relationships that could be construed as a potential conflict of interest.

## References

[B1] Akin B. L. Hurley T. D. Chen Z. Jones L. R. (2013). The structural basis for phospholamban inhibition of the calcium pump in sarcoplasmic reticulum. J. Biol. Chem. 288, 30181–30191. 10.1074/jbc.M113.501585 23996003 PMC3798486

[B2] Andersen J. P. (1995). Functional consequences of alterations to amino acids at the M5S5 boundary of the Ca(2+)-ATPase of sarcoplasmic reticulum. Mutation Tyr763–>Gly uncouples ATP hydrolysis from Ca(2+) transport. J. Biol. Chem. 270, 908–914. 10.1074/jbc.270.2.908 7822330

[B3] Andersen J. P. Vilsen B. (1992). Functional consequences of alterations to Glu309, Glu771, and Asp800 in the Ca(2+)-ATPase of sarcoplasmic reticulum. J. Biol. Chem. 267, 19383–19387. 10.1016/S0021-9258(18)41787-5 1388169

[B4] Asahi M. Kimura Y. Kurzydlowski K. Tada M. MacLennan D. H. (1999). Transmembrane helix M6 in sarco(endo)plasmic reticulum Ca(2+)-ATPase forms a functional interaction site with phospholamban. Evidence for physical interactions at other sites. J. Biol. Chem. 274, 32855–32862. 10.1074/jbc.274.46.32855 10551848

[B5] Asahi M. Kurzydlowski K. Tada M. MacLennan D. H. (2002). Sarcolipin inhibits polymerization of phospholamban to induce superinhibition of sarco(endo)plasmic reticulum Ca(2+)-ATPases (SERCAs). J. Biol. Chem. 277, 26725–26728. 10.1074/jbc.C200269200 12032137

[B6] Asahi M. Sugita Y. Kurzydlowski K. De Leon S. Tada M. Toyoshima C.. (2003). Sarcolipin regulates sarco(endo)plasmic reticulum Ca(2+)-ATPase (SERCA) by binding to transmembrane helices alone or in association with phospholamban. Proc. Natl. Acad. Sci. U.S.A. 100, 5040–5045. 10.1073/pnas.0330962100 12692302 PMC154294

[B7] Autry J. M. Thomas D. D. Espinoza-Fonseca L. M. (2016). Sarcolipin promotes uncoupling of the SERCA Ca(2+) pump by inducing a structural rearrangement in the energy-transduction domain. Biochemistry 55, 6083–6086. 10.1021/acs.biochem.6b00728 27731980 PMC5506494

[B8] Barbot T. Montigny C. Decottignies P. Le Maire M. Jaxel C. Jamin N.. (2016). “Functional and structural insights into sarcolipin, a regulator of the sarco-endoplasmic reticulum Ca(2+)-ATPases,” in Regulation of Ca(2+)-ATPases,V-ATPases and F-ATPases, eds S. Chakraborti and N. S. Dhalla (Cham: Springer), 153–186.

[B9] Best R. B. Zhu X. Shim J. Lopes P. E. M. Mittal J. Feig M.. (2012). Optimization of the additive CHARMM all-atom protein force field targeting improved sampling of the backbone φ, ψ and side-chain χ1 and χ2 dihedral angles. J. Chem. Theory Comput. 8, 3257–3273. 10.1021/ct300400x 23341755 PMC3549273

[B10] Bick R. J. Buja L. M. Van Winkle W. B. Taffet G. E. (1998). Membrane asymmetry in isolated canine cardiac sarcoplasmic reticulum: comparison with skeletal muscle sarcoplasmic reticulum. J. Membr. Biol. 164, 169–175. 10.1007/s002329900402 9662560

[B11] Brooks B. R. Bruccoleri R. E. Olafson B. D. States D. J. Swaminathan S. Karplus M. (1983). CHARMM: a program for macromolecular energy, minimization, and dynamics calculations. J. Comput. Chem. 4, 187–217. 10.1002/jcc.540040211

[B12] Buffy J. J. Buck-Koehntop B. A. Porcelli F. Traaseth N. J. Thomas D. D. Veglia G. (2006). Defining the intramembrane binding mechanism of sarcolipin to calcium ATPase using solution NMR spectroscopy. J. Mol. Biol. 358, 420–429. 10.1016/j.jmb.2006.02.005 16519897

[B13] Chen V. B. Arendall W. B. Headd J. J. Keedy D. A. Immormino R. M. Kapral G. J.. (2010). *MolProbity* : all-atom structure validation for macromolecular crystallography. Acta Crystallogr. D Biol. Crystallogr. 66, 12–21. 10.1107/S0907444909042073 20057044 PMC2803126

[B14] Chen Z. (2015). Role of nucleotides in stabilization of the phospholamban/cardiac Ca(2+) pump inhibitory complex examined with use of metal fluorides. FEBS J. 282, 4402–4414. 10.1111/febs.13506 26337774

[B15] Clarke D. M. Loo T. W. Inesi G. MacLennan D. H. (1989). Location of high affinity Ca(2+)-binding sites within the predicted transmembrane domain of the sarcoplasmic reticulum Ca(2+)-ATPase. Nature 339, 476–478. 10.1038/339476a0 2524669

[B16] Clausen J. D. Bublitz M. Arnou B. Montigny C. Jaxel C. Møller J. V.. (2013). SERCA mutant E309Q binds two Ca(2+) ions but adopts a catalytically incompetent conformation. EMBO J. 32, 3231–3243. 10.1038/emboj.2013.250 24270570 PMC3981149

[B17] Clausen J. D. Bublitz M. Arnou B. Olesen C. Andersen J. P. Møller J. V.. (2016). Crystal structure of the vanadate-inhibited Ca(2+)-ATPase. Struct. Lond. Engl. 24, 617–623. 10.1016/j.str.2016.02.018 27050689

[B18] Clausen J. D. McIntosh D. B. Woolley D. G. Andersen J. P. (2001). Importance of Thr-353 of the conserved phosphorylation loop of the sarcoplasmic reticulum Ca(2+)-ATPase in MgATP binding and catalytic activity. J. Biol. Chem. 276, 35741–35750. 10.1074/jbc.M105434200 11438551

[B19] Douglas J. L. Trieber C. A. Afara M. Young H. S. (2005). Rapid, high-yield expression and purification of Ca(2+)-ATPase regulatory proteins for high-resolution structural studies. Protein Expr. Purif. 40, 118–125. 10.1016/j.pep.2004.11.015 15721779

[B20] Ebashi S. Lipmann F. (1962). Adenosine triphosphate-linked concentration of calcium ions in a particulate fraction of rabbit muscle. J. Cell Biol. 14, 389–400. 10.1083/jcb.14.3.389 19866605 PMC2106118

[B21] Espinoza-Fonseca L. M. Autry J. M. Ramírez-Salinas G. L. Thomas D. D. (2015a). Atomic-level mechanisms for phospholamban regulation of the calcium pump. Biophys. J. 108, 1697–1708. 10.1016/j.bpj.2015.03.004 25863061 PMC4390807

[B22] Espinoza-Fonseca L. M. Autry J. M. Thomas D. D. (2014). Microsecond molecular dynamics simulations of Mg2+- and K+- bound E1 intermediate states of the calcium pump. PLOS ONE 9:e95979. 10.1371/journal.pone.0095979 24760008 PMC3997511

[B23] Espinoza-Fonseca L. M. Autry J. M. Thomas D. D. (2015b). Sarcolipin and phospholamban inhibit the calcium pump by populating a similar metal ion-free intermediate state. Biochem. Biophys. Res. Commun. 463, 37–41. 10.1016/j.bbrc.2015.05.012 25983321 PMC4465059

[B24] Falson P. Menguy T. Corre F. Bouneau L. de Gracia A. G. Souli,é S.. (1997). The cytoplasmic loop between putative transmembrane segments 6 and 7 in sarcoplasmic reticulum Ca(2+)-ATPase binds Ca(2+) and is functionally important. J. Biol. Chem. 272, 17258–17262. 10.1074/jbc.272.28.17258 9211861

[B25] Fernández-de Gortari E. Espinoza-Fonseca L. M. (2018). Structural basis for relief of phospholamban-mediated inhibition of the sarcoplasmic reticulum Ca(2+)-ATPase at saturating Ca(2+) conditions. J. Biol. Chem. 293, 12405–12414. 10.1074/jbc.RA118.003752 29934304 PMC6093236

[B26] Galtier N. Gouy M. Gautier C. (1996). SEAVIEW and PHYLO_WIN: two graphic tools for sequence alignment and molecular phylogeny. Comput. Appl. Biosci. CABIOS 12, 543–548. 10.1093/bioinformatics/12.6.543 9021275

[B27] Gorski P. A. Glaves J. P. Vangheluwe P. Young H. S. (2013). Sarco(endo)plasmic reticulum calcium ATPase (SERCA) inhibition by sarcolipin is encoded in its luminal tail. J. Biol. Chem. 288, 8456–8467. 10.1074/jbc.M112.446161 23362265 PMC3605661

[B28] Gould G. W. McWhirter J. M. East J. M. Lee A. G. (1987). Effects of diet on the function of sarcoplasmic reticulum. Biochem. J. 245, 751–755. 10.1042/bj2450751 2959280 PMC1148194

[B29] Guindon S. Dufayard J.-F. Lefort V. Anisimova M. Hordijk W. Gascuel O. (2010). New algorithms and methods to estimate maximum-likelihood phylogenies: assessing the performance of PhyML 3.0. Syst. Biol. 59, 307–321. 10.1093/sysbio/syq010 20525638

[B30] Hasselbach W. Makinose M. (1961). The calcium pump of the “relaxing granules” of muscle and its dependence on ATP-splitting. Biochem. Z. 333, 518–528. 13712164

[B31] Hughes E. Clayton J. C. Kitmitto A. Esmann M. Middleton D. A. (2007). Solid-state NMR and functional measurements indicate that the conserved tyrosine residues of sarcolipin are involved directly in the inhibition of SERCA1. J. Biol. Chem. 282, 26603–26613. 10.1074/jbc.M611668200 17616528

[B32] Hughes E. Middleton D. A. (2003). Solid-state NMR reveals structural changes in phospholamban accompanying the functional regulation of Ca(2+)-ATPase. J. Biol. Chem. 278, 20835–20842. 10.1074/jbc.M212208200 12556441

[B33] Humphrey W. Dalke A. Schulten K. (1996). VMD: visual molecular dynamics. J. Mol. Graph. 14, 33–38, 27–28. 10.1016/0263-7855(96)00018-5 8744570

[B34] Inesi G. Zhang Z. Lewis D. (2002). Cooperative setting for long-range linkage of Ca(2+) binding and ATP synthesis in the Ca(2+) ATPase. Biophys. J. 83, 2327–2332. 10.1016/S0006-3495(02)75247-8 12414670 PMC1302322

[B35] Jo S. Kim T. Im W. (2007). Automated builder and database of protein/membrane complexes for molecular dynamics simulations. PLoS ONE 2:e880. 10.1371/journal.pone.0000880 17849009 PMC1963319

[B36] Jo S. Kim T. Iyer V. G. Im W. (2008). CHARMM-GUI: a web-based graphical user interface for CHARMM. J. Comput. Chem. 29, 1859–1865. 10.1002/jcc.20945 18351591

[B37] Jo S. Lim J. B. Klauda J. B. Im W. (2009). CHARMM-GUI membrane builder for mixed bilayers and its application to yeast membranes. Biophys. J. 97, 50–58. 10.1016/j.bpj.2009.04.013 19580743 PMC2711372

[B38] Jorgensen W. L. Chandrasekhar J. Madura J. D. Impey R. W. Klein M. L. (1983). Comparison of simple potential functions for simulating liquid water. J. Chem. Phys. 79, 926–935. 10.1063/1.445869

[B39] Kirchberber M. A. Tada M. Katz A. M. (1975). Phospholamban: a regulatory protein of the cardiac sarcoplasmic reticulum. Recent Adv. Stud. Cardiac Struct. Metab. 5, 103–115. 127351

[B40] Klauda J. B. Venable R. M. Freites J. A. O'Connor J. W. Tobias D. J. Mondragon-Ramirez C.. (2010). Update of the CHARMM all-atom additive force field for lipids: validation on six lipid types. J. Phys. Chem. B 114, 7830–7843. 10.1021/jp101759q 20496934 PMC2922408

[B41] Lenoir G. Jaxel C. Picard M. le Maire M. Champeil P. Falson P. (2006). Conformational changes in sarcoplasmic reticulum Ca(2+)-ATPase mutants: effect of mutations either at Ca(2+)-binding site II or at tryptophan 552 in the cytosolic domain. Biochemistry 45, 5261–5270. 10.1021/bi0522091 16618114

[B42] Li G. Cui Q. (2002). A coarse-grained normal mode approach for macromolecules: an efficient implementation and application to Ca(2+)-ATPase. Biophys. J. 83, 2457–2474. 10.1016/S0006-3495(02)75257-0 12414680 PMC1302332

[B43] Lomize M. A. Pogozheva I. D. Joo H. Mosberg H. I. Lomize A. L. (2012). OPM database and PPM web server: resources for positioning of proteins in membranes. Nucleic Acids Res. 40, D370–D376. 10.1093/nar/gkr703 21890895 PMC3245162

[B44] Ma H. Inesi G. Toyoshima C. (2003). Substrate-induced conformational fit and headpiece closure in the Ca(2+)ATPase (SERCA). J. Biol. Chem. 278, 28938–28943. 10.1074/jbc.M304120200 12750373

[B45] Ma H. Lewis D. Xu C. Inesi G. Toyoshima C. (2005). Functional and structural roles of critical amino acids within the “N”, “P”, and “A” domains of the Ca(2+) ATPase (SERCA) headpiece. Biochemistry 44, 8090–8100. 10.1021/bi050332m 15924428

[B46] MacLennan D. H. Asahi M. Tupling A. R. (2003). The regulation of SERCA-type pumps by phospholamban and sarcolipin. Ann. N. Y. Acad. Sci. 986, 472–480. 10.1111/j.1749-6632.2003.tb07231.x 12763867

[B47] MacLennan D. H. Brandl C. J. Korczak B. Green N. M. (1985). Amino-acid sequence of a Ca(2+) + Mg2+-dependent ATPase from rabbit muscle sarcoplasmic reticulum, deduced from its complementary DNA sequence. Nature 316, 696–700. 10.1038/316696a0 2993904

[B48] MacLennan D. H. Kranias E. G. (2003). Phospholamban: a crucial regulator of cardiac contractility. Nat. Rev. Mol. Cell Biol. 4, 566–577. 10.1038/nrm1151 12838339

[B49] Maruyama K. Clarke D. M. Fujii J. Inesi G. Loo T. W. MacLennan D. H. (1989). Functional consequences of alterations to amino acids located in the catalytic center (isoleucine 348 to threonine 357) and nucleotide-binding domain of the Ca(2+)-ATPase of sarcoplasmic reticulum. J. Biol. Chem. 264, 13038–13042. 10.1016/S0021-9258(18)51592-1 2526811

[B50] Menguy T. Corre F. Bouneau L. Deschamps S. Møller J. V. Champeil P.. (1998). The cytoplasmic loop located between transmembrane segments 6 and 7 controls activation by Ca(2+) of sarcoplasmic reticulum Ca(2+)-ATPase. J. Biol. Chem. 273, 20134–20143. 10.1074/jbc.273.32.20134 9685357

[B51] Menguy T. Corre F. Juul B. Bouneau L. Lafitte D. Derrick P. J.. (2002). Involvement of the cytoplasmic loop L6-7 in the entry mechanism for transport of Ca(2+) through the sarcoplasmic reticulum Ca(2+)-ATPase. J. Biol. Chem. 277, 13016–13028. 10.1074/jbc.M108899200 11801592

[B52] Møller J. V. Juul B. le Maire M. (1996). Structural organization, ion transport, and energy transduction of P-type ATPases. Biochim. Biophys. Acta 1286, 1–51. 10.1016/0304-4157(95)00017-8 8634322

[B53] Møller J. V. Olesen C. Winther A.-M. L. Nissen P. (2010). The sarcoplasmic Ca(2+)-ATPase: design of a perfect chemi-osmotic pump. Q. Rev. Biophys. 43, 501–566. 10.1017/S003358351000017X 20809990

[B54] Montigny C. Huang D. H. Beswick V. Barbot T. Jaxel C. le Maire M.. (2021). Sarcolipin alters SERCA1a interdomain communication by impairing binding of both calcium and ATP. Sci. Rep. 11:1641. 10.1038/s41598-021-81061-6 33452371 PMC7810697

[B55] Montigny C. Picard M. Lenoir G. Gauron C. Toyoshima C. Champeil P. (2007). Inhibitors bound to Ca(2+)-free sarcoplasmic reticulum Ca(2+)-ATPase lock its transmembrane region but not necessarily its cytosolic region, revealing the flexibility of the loops connecting transmembrane and cytosolic domains. Biochemistry 46, 15162–15174. 10.1021/bi701855r 18052080

[B56] Mouawad L. Perahia D. (1993). Diagonalization in a mixed basis: a method to compute low-frequency normal modes for large macromolecules. Biopolymers 33, 599–611. 10.1002/bip.360330409 7551557

[B57] Neria E. Fischer S. Karplus M. (1996). Simulation of activation free energies in molecular systems. J. Chem. Phys. 105, 1902–1921. 10.1063/1.472061

[B58] Odermatt A. Becker S. Khanna V. K. Kurzydlowski K. Leisner E. Pette D.. (1998). Sarcolipin regulates the activity of SERCA1, the fast-twitch skeletal muscle sarcoplasmic reticulum Ca(2+)-ATPase. J. Biol. Chem. 273, 12360–12369. 10.1074/jbc.273.20.12360 9575189

[B59] Odermatt A. Taschner P. E. Scherer S. W. Beatty B. Khanna V. K. Cornblath D. R.. (1997). Characterization of the gene encoding human sarcolipin (SLN), a proteolipid associated with SERCA1: absence of structural mutations in five patients with Brody disease. Genomics 45, 541–553. 10.1006/geno.1997.4967 9367679

[B60] Olesen C. Picard M. Winther A.-M. L. Gyrup C. Morth J. P. Oxvig C.. (2007). The structural basis of calcium transport by the calcium pump. Nature 450, 1036–1042. 10.1038/nature06418 18075584

[B61] Orlowski S. Champeil P. (1991a). Kinetics of calcium dissociation from its high-affinity transport sites on sarcoplasmic reticulum ATPase. Biochemistry 30, 352–361. 10.1021/bi00216a007 1824819

[B62] Orlowski S. Champeil P. (1991b). The two calcium ions initially bound to nonphosphorylated sarcoplasmic reticulum Ca(2+)-ATPase can no longer be kinetically distinguished when they dissociate from phosphorylated ATPase toward the lumen. Biochemistry 30, 11331–11342. 10.1021/bi00111a020 1835657

[B63] Perahia D. Mouawad L. (1995). Computation of low-frequency normal modes in macromolecules: improvements to the method of diagonalization in a mixed basis and application to hemoglobin. Comput. Chem. 19, 241–246. 10.1016/0097-8485(95)00011-G 7551557

[B64] Picard M. Toyoshima C. Champeil P. (2006). Effects of inhibitors on luminal opening of Ca(2+) binding sites in an E2P-like complex of sarcoplasmic reticulum Ca22+-ATPase with Be22+-fluoride. J. Biol. Chem. 281, 3360–3369. 10.1074/jbc.M511385200 16332689

[B65] Reuter N. Hinsen K. Lacapère J.-J. (2003). Transconformations of the SERCA1 Ca-ATPase: a normal mode study. Biophys. J. 85, 2186–2197. 10.1016/S0006-3495(03)74644-X 14507684 PMC1303445

[B66] Rice W. J. MacLennan D. H. (1996). Scanning mutagenesis reveals a similar pattern of mutation sensitivity in transmembrane sequences M4, M5, and M6, but not in M8, of the Ca(2+)-ATPase of sarcoplasmic reticulum (SERCA1a). J. Biol. Chem. 271, 31412–31419. 10.1074/jbc.271.49.31412 8940151

[B67] Ridder I. S. Dijkstra B. W. (1999). Identification of the Mg2+-binding site in the P-type ATPase and phosphatase members of the HAD (haloacid dehalogenase) superfamily by structural similarity to the response regulator protein CheY. Biochem. J. 339(Pt. 2), 223–226. 10.1042/bj3390223 10191250 PMC1220148

[B68] Sacchetto R. Bertipaglia I. Giannetti S. Cendron L. Mascarello F. Damiani E.. (2012). Crystal structure of sarcoplasmic reticulum Ca(2+)-ATPase (SERCA) from bovine muscle. J. Struct. Biol. 178, 38–44. 10.1016/j.jsb.2012.02.008 22387132

[B69] Sahoo S. K. Shaikh S. A. Sopariwala D. H. Bal N. C. Bruhn D. S. Kopec W.. (2015). The N terminus of sarcolipin plays an important role in uncoupling sarco-endoplasmic reticulum Ca(2+)-ATPase (SERCA) ATP hydrolysis from Ca(2+) transport. J. Biol. Chem. 290, 14057–14067. 10.1074/jbc.M115.636738 25882845 PMC4447977

[B70] Sahoo S. K. Shaikh S. A. Sopariwala D. H. Bal N. C. Periasamy M. (2013). Sarcolipin protein interaction with sarco(endo)plasmic reticulum Ca(2+) ATPase (SERCA) is distinct from phospholamban protein, and only sarcolipin can promote uncoupling of the SERCA pump. J. Biol. Chem. 288, 6881–6889. 10.1074/jbc.M112.436915 23341466 PMC3591597

[B71] Smith W. S. Broadbridge R. East J. M. Lee A. G. (2002). Sarcolipin uncouples hydrolysis of ATP from accumulation of Ca(2+) by the Ca(2+)-ATPase of skeletal-muscle sarcoplasmic reticulum. Biochem. J. 361, 277–286. 10.1042/bj3610277 11772399 PMC1222307

[B72] Sorensen T. L. Andersen J. P. (2000). Importance of stalk segment S5 for intramolecular communication in the sarcoplasmic reticulum Ca(2+)-ATPase. J. Biol. Chem. 275, 28954–28961. 10.1074/jbc.M004072200 10871620

[B73] Sørensen T. L.-M. Møller J. V. Nissen P. (2004). Phosphoryl transfer and calcium ion occlusion in the calcium pump. Science 304, 1672–1675. 10.1126/science.1099366 15192230

[B74] Soulié S. Neumann J. M. Berthomieu C. Møller J. V. le Maire M. Forge V. (1999). NMR conformational study of the sixth transmembrane segment of sarcoplasmic reticulum Ca(2+)-ATPase. Biochemistry 38, 5813–5821. 10.1021/bi983039d 10231532

[B75] Starling A. P. East J. M. Lee A. G. (1993). Effects of phosphatidylcholine fatty acyl chain length on calcium binding and other functions of the (Ca(2+)-Mg2+)-ATPase. Biochemistry 32, 1593–1600. 10.1021/bi00057a025 8431438

[B76] Toyoshima C. (2009). How Ca(2+)-ATPase pumps ions across the sarcoplasmic reticulum membrane. Biochim. Biophys. Acta 1793, 941–946. 10.1016/j.bbamcr.2008.10.008 19010358

[B77] Toyoshima C. Iwasawa S. Ogawa H. Hirata A. Tsueda J. Inesi G. (2013). Crystal structures of the calcium pump and sarcolipin in the Mg2+-bound E1 state. Nature 495, 260–264. 10.1038/nature11899 23455422

[B78] Toyoshima C. Mizutani T. (2004). Crystal structure of the calcium pump with a bound ATP analogue. Nature 430, 529–535. 10.1038/nature02680 15229613

[B79] Toyoshima C. Nomura H. Tsuda T. (2004). Lumenal gating mechanism revealed in calcium pump crystal structures with phosphate analogues. Nature 432, 361–368. 10.1038/nature02981 15448704

[B80] Toyoshima C. Yonekura S.-I. Tsueda J. Iwasawa S. (2011). Trinitrophenyl derivatives bind differently from parent adenine nucleotides to Ca(2+)-ATPase in the absence of Ca(2+). Proc. Natl. Acad. Sci. U.S.A. 108, 1833–1838. 10.1073/pnas.1017659108 21239683 PMC3033254

[B81] Tupling A. R. Asahi M. MacLennan D. H. (2002). Sarcolipin overexpression in rat slow twitch muscle inhibits sarcoplasmic reticulum Ca(2+) uptake and impairs contractile function. J. Biol. Chem. 277, 44740–44746. 10.1074/jbc.M206171200 12237298

[B82] Vilsen B. Andersen J. P. (1992). Mutational analysis of the role of Glu309 in the sarcoplasmic reticulum Ca(2+)-ATPase of frog skeletal muscle. FEBS Lett. 306, 247–250. 10.1016/0014-5793(92)81010-J 1386028

[B83] Winther A.-M. L. Bublitz M. Karlsen J. L. Møller J. V. Hansen J. B. Nissen P.. (2013). The sarcolipin-bound calcium pump stabilizes calcium sites exposed to the cytoplasm. Nature 495, 265–269. 10.1038/nature11900 23455424

[B84] Wu E. L. Cheng X. Jo S. Rui H. Song K. C. Dávila-Contreras E. M.. (2014). CHARMM-GUI membrane builder toward realistic biological membrane simulations. J. Comput. Chem. 35, 1997–2004. 10.1002/jcc.23702 25130509 PMC4165794

[B85] Zhang P. Toyoshima C. Yonekura K. Green N. M. Stokes D. L. (1998). Structure of the calcium pump from sarcoplasmic reticulum at 8-A resolution. Nature 392, 835–839. 10.1038/33959 9572145

[B86] Zhang Z. Lewis D. Strock C. Inesi G. Nakasako M. Nomura H.. (2000). Detailed characterization of the cooperative mechanism of Ca(2+) binding and catalytic activation in the Ca(2+) transport (SERCA) ATPase. Biochemistry 39, 8758–8767. 10.1021/bi000185m 10913287

