# Corrigendum: Deciphering the mechanism of inhibition of SERCA1a by sarcolipin using molecular simulations

**DOI:** 10.3389/fmolb.2022.1035445

**Published:** 2022-10-13

**Authors:** Thomas Barbot, Veronica Beswick, Cédric Montigny, Éric Quiniou, Nadège Jamin, Liliane Mouawad

**Affiliations:** ^1^ Institute for Integrative Biology of the Cell (I2BC), CEA, CNRS, Université Paris-Saclay, Gif-sur-Yvette, France; ^2^ Physics Department, Evry-Val-d’Essonne University, Paris-Saclay University, Evry, France; ^3^ CNRS UMR9187 / INSERM U1196, Institut Curie, PSL Research University, Université Paris-Saclay, Orsay, France

**Keywords:** normal mode analysis, molecular simulations, molecular modeling, calcium ATPase, SERCA1a, sarcolipin

In the published article, there was an error in the equation for atomic fluctuations. A correction has been made to **Methods, Calculations, Atomic fluctuations**, paragraph 1. The corrected sentence appears below:

“For each structure, E1.Mg^2+^:SLN and E1.Mg^2+^, the atomic fluctuations, $f_{i}$ , were calculated from the 200 modes using:

$$f_{i}=\sqrt{\langle \Delta {\vec{r}}_{i}^{2}\rangle }=\sqrt{k_{B}T\overset{l}{\underset{m=7}{\sum }}\frac{{\vec{q}}_{im}^{2}}{\omega_{m}^{2}}}$$

”

The authors apologize for this error and state that this does not change the scientific conclusions of the article in any way. The original article has been updated.

