# Supplementary material for: Deciphering the Mechanism of Inhibition of SERCA1a by Sarcolipin Using Molecular Simulations
Source: Front Mol Biosci. 2021 Feb 4;7:606254. doi: 10.3389/fmolb.2020.606254 (PMC7890198; doi:10.3389/fmolb.2020.606254)
Supplement: Supplementary file 1 [file Presentation_1.pdf]

SUPPLEMENTARY MATERIAL

Deciphering the mechanism of inhibition of SERCA1a by sarcolipin using molecular simulations

Thomas Barbot, Veronica Beswick*, Cédric Montigny, Éric Quiniou, Nadège Jamin and Liliane Mouawad*

CONTENTS

Table S1. A non-exhaustive list of the crystal structures of SERCA1a

Figure S1. Comparison of the SERCA1a structures in the E1.2Ca^{2+}, E1.Mg^{2+}:SLN, and E2 states

Table S2. Assessment of the quality of the structures

Figure S2. Assessment of the quality of the structures

Figure S3. Motions along the two modes identified from the modes/mode overlaps

Figure S4. Fluctuations of the C_α atoms

Figure S5. Difference of fluctuations of the C_α atoms

Table S3. Distances in the metal binding sites

Figure S6. Comparison of the difference between E1.Mg^{2+} and E1.Mg^{2+}:SLN for the energy-minimized structures and the crystal structures

Figure S7. Zoom on the C-terminal tail of SLN

References

Table S1: A non-exhaustive list of the crystal structures of SERCA1a. Refences of the crystal structures are at the end of the Supplementary file.

State	Organism	PDB ID	Ion(s) in the Ca^{2+}-binding sites	ADP or ATP analogues	Other molecules	Commentary
**E1.2Ca^{2+}**	Oryctolagus Cuniculus	1VFP[1]	2 Ca^{2+}	AMPPCP	None	Resolution : 2.90 Å
	Oryctolagus Cuniculus	3AR2[2]	2 Ca^{2+}	AMPPCP	None	Resolution : 2.50 Å<br>ATP γ-phosphate chelates Ca^{2+} instead of Mg^{2+}
	Oryctolagus Cuniculus	1T5S[3]	2 Ca^{2+}	AMPPCP	None	Resolution : 2.60 Å<br>Lacks the SS-bridge
	Bos Taurus	3TLM[4]	2 Ca^{2+}	AMPPCP	None	Resolution : 2.95 Å<br>Missing residues<br>Lacks the SS-bridge
**E1~P.2Ca^{2+}**	Oryctolagus Cuniculus	2ZBD[5]	2 Ca^{2+}	ADP	AlF_4	Resolution : 2.40 Å
	Oryctolagus Cuniculus	1T5T[3]	2 Ca^{2+}	ADP	AlF_4	Resolution : 2.90 Å
**E2P.2Ca^{2+}**	Oryctolagus Cuniculus	5A3R[6]	1 Mg^{2+}	TNPAMPPCP	BeF_3	Resolution : 3.05 Å
	Oryctolagus Cuniculus	3B9B[7]	1 Mg^{2+}	None	BeF_3	Resolution : 2.65 Å
**E2**	Oryctolagus Cuniculus	3W5C[8]	None	None	None	Resolution : 2.50 Å
	Oryctolagus Cuniculus	2DQS[9]	None	AMPPCP	Thapsigargin	Resolution : 2.50 Å
**E1.Mg^{2+}**	Oryctolagus Cuniculus	3W5B[8]	1 Mg^{2+}	TNPAMP	None	Resolution : 3.20 Å<br>Expressed in Chlorocebus Sabaeus cells
**E1:PLN**	Oryctolagus Cuniculus	4KYT[10]	None	None	Phospholamban	Resolution : 2.83 Å<br>Missing residues<br>Expressed in Spodoptera Frugiperda cells
**E1.Mg^{2+}:SLN**	Oryctolagus Cuniculus	3W5A[8]	1 Mg^{2+}	TNPAMP	Sarcolipin	Resolution : 3.01 Å
**E1.2Mg^{2+}:SLN**	Oryctolagus Cuniculus	4H1W[11]	2 Mg^{2+}	AMPPCP	Sarcolipin	Resolution : 3.10 Å<br>Missing residues

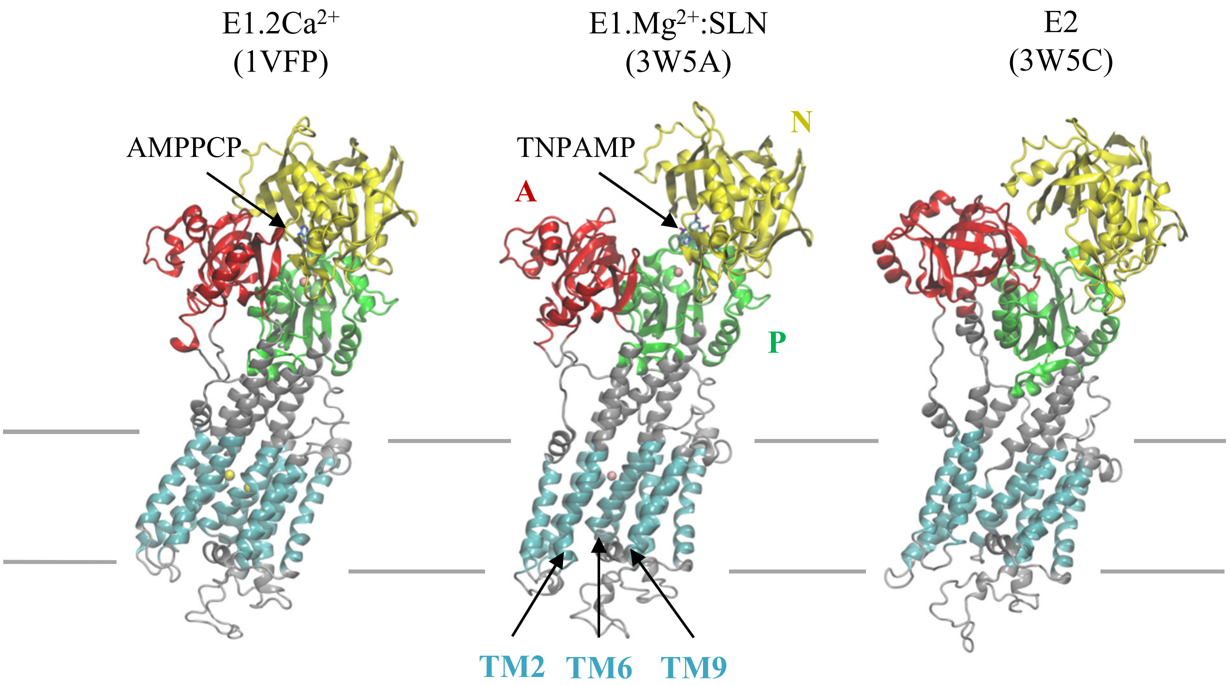

Figure S1. Comparison of the SERCA1a crystal structures in the E1.2Ca^{2+}, E1.Mg^{2+}:SLN, and E2 states. The color code is the same as in Figure 1A in the main text, i.e., the N-, P- and A-domains are in yellow, green and red, respectively, the TM domain in cyan and the rest of the protein in gray. The ATP analogs are in sticks colored according to atom names, C: cyan, N: blue, O: red, P: tan and Mg^{2+}: pink. The three structures are oriented in the same way, in order to have the SLN binding groove, formed by helices TM2, TM6 and TM9, facing the reader. In the structure E1.Mg^{2+}:SLN, SLN was omitted to allow the reader to see TM6 and the bound Mg^{2+} (pink sphere), which otherwise would be hidden by the peptide. In E1.2Ca^{2+}, calcium ions are presented as yellow spheres. Note that here, exceptionnally, E1.Mg^{2+}:SLN does not refer to the energy-minimized structure, but to the state of the crystal structure 3W5A.

Table S2: Assessment of the quality of the structures. The quality of 3W5A – chain B, the crystal structure of SERCA1a in the presence of SLN (first column), the three energy-minimized structures in the presence of SLN (columns 2, 3, and 4) and in its absence (column 5) was evaluated using the Swissmodel website. For a good quality structure, the MolProbity score, the clash score, the percentage of Ramachandran outliers, and that of the rotamer outliers should be as small as possible, whereas the percentage of residues in the Ramachandran favored regions should be more than 90%.

	3W5A–chain B (SERCA1a with SLN)	E1.Mg^{2+}:SLN (1)	E1.Mg^{2+}:SLN (2)	E1.Mg^{2+}:SLN (3)	E1.Mg^{2+}
**MolProbity Score**	2.93	1.32	1.33	1.43	1.40
**Clash Score**	12.74	0.13	0.33	0.40	0.07
**Ramachandran Favored**	85.59%	91.43%	91.43%	91.83%	90.93%
**Ramachandran Outliers**	2.02%	0.50%	1.01%	1.01%	0.30%
**Rotamer Outliers**	7.94%	2.21%	1.82%	2.47%	2.86%

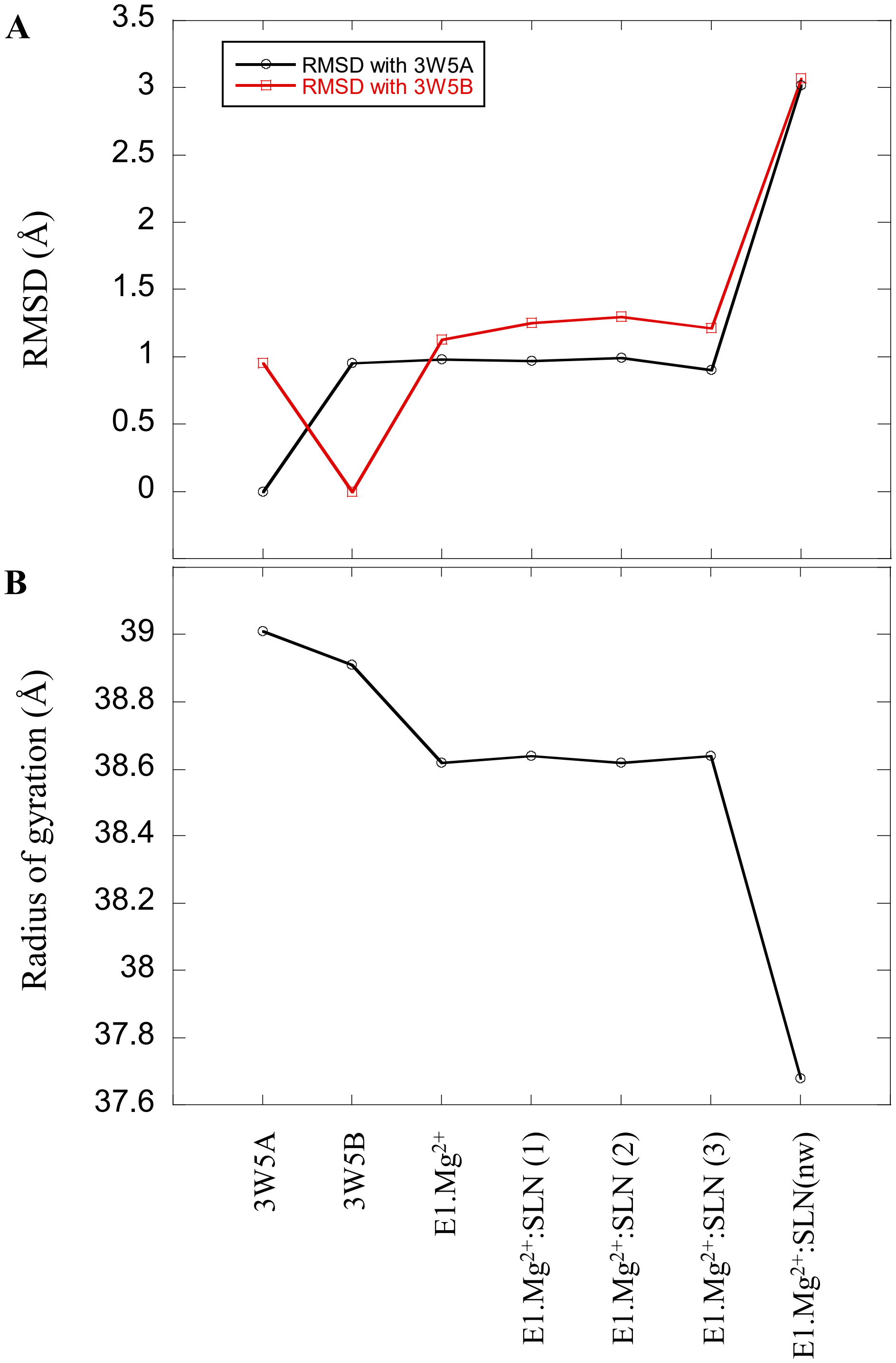

Figure S2. Assessment of the quality of the structures. (A) RMSD of C_α atoms with respect to the crystal structures of SERCA1a-SLN (3W5A) in black and SERCA1a (3W5B) in red of the two crystal structures, and the energy minimized structures in the absence of SLN (E1.Mg^{2+}) and its presence. In the presence of SLN, there are 3 structures with one water shell and the structure with no water (nw). (B) The radius of gyration of all the cited structures, calculated for only SERCA1a where all elements that are not in common were removed for the sake of comparison, i.e., SLN, ATP or ATP analogs, and hydrogen atoms.

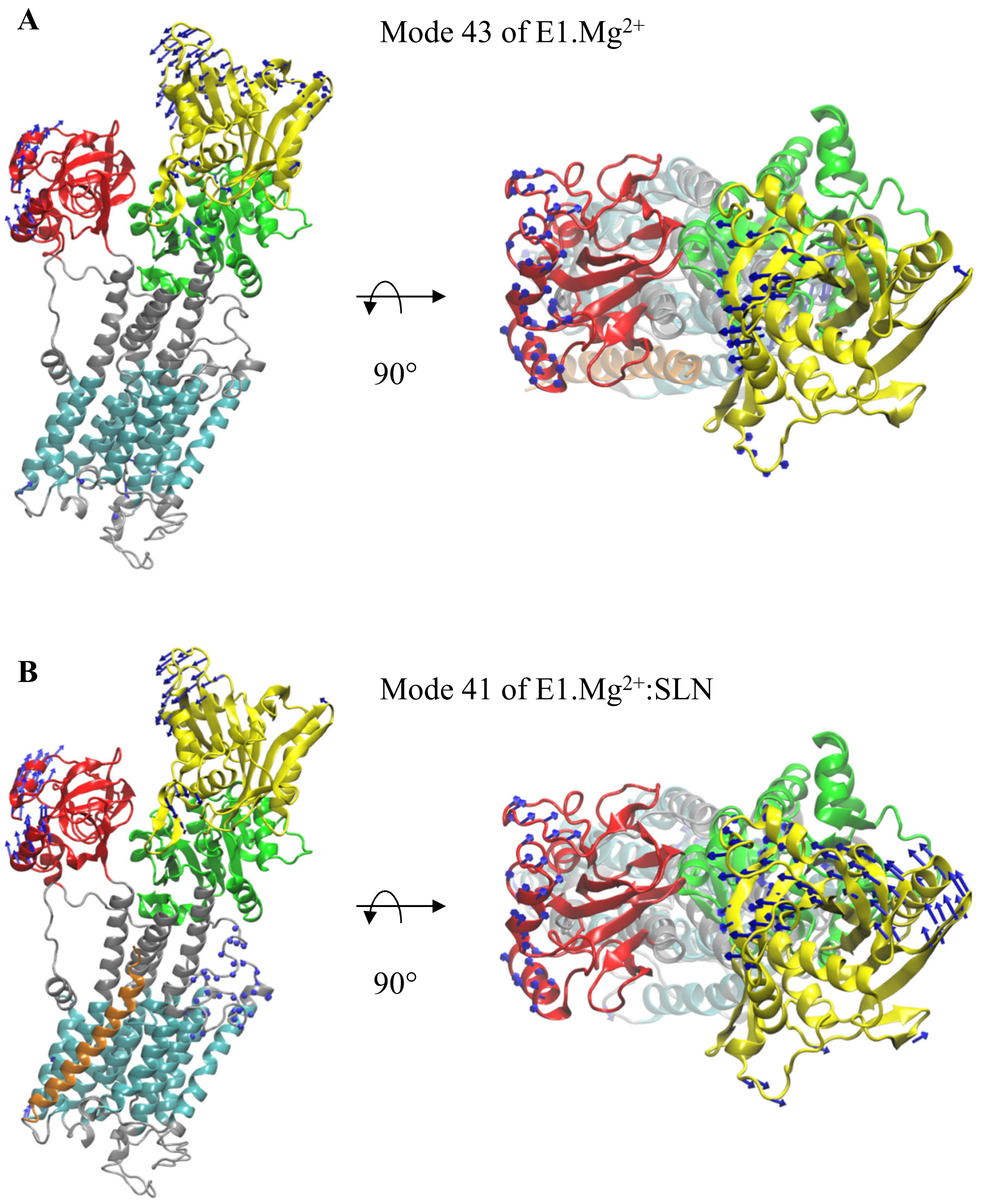

Figure S3. Motions along the two modes identified from the modes/mode overlaps. In each panel a profile and a top view of the protein are presented. The same color code as in Figure 1A in the main text is used. The directions of motions are shown as blue arrows and the lengths of the arrows are proportional to the amplitude of displacement. For clarity, the arrows corresponding to displacement amplitudes under 3 Å are omitted. The motions are shown along mode 43 of E1.Mg^{2+} (A) and mode 41 of E1.Mg^{2+}:SLN (B).

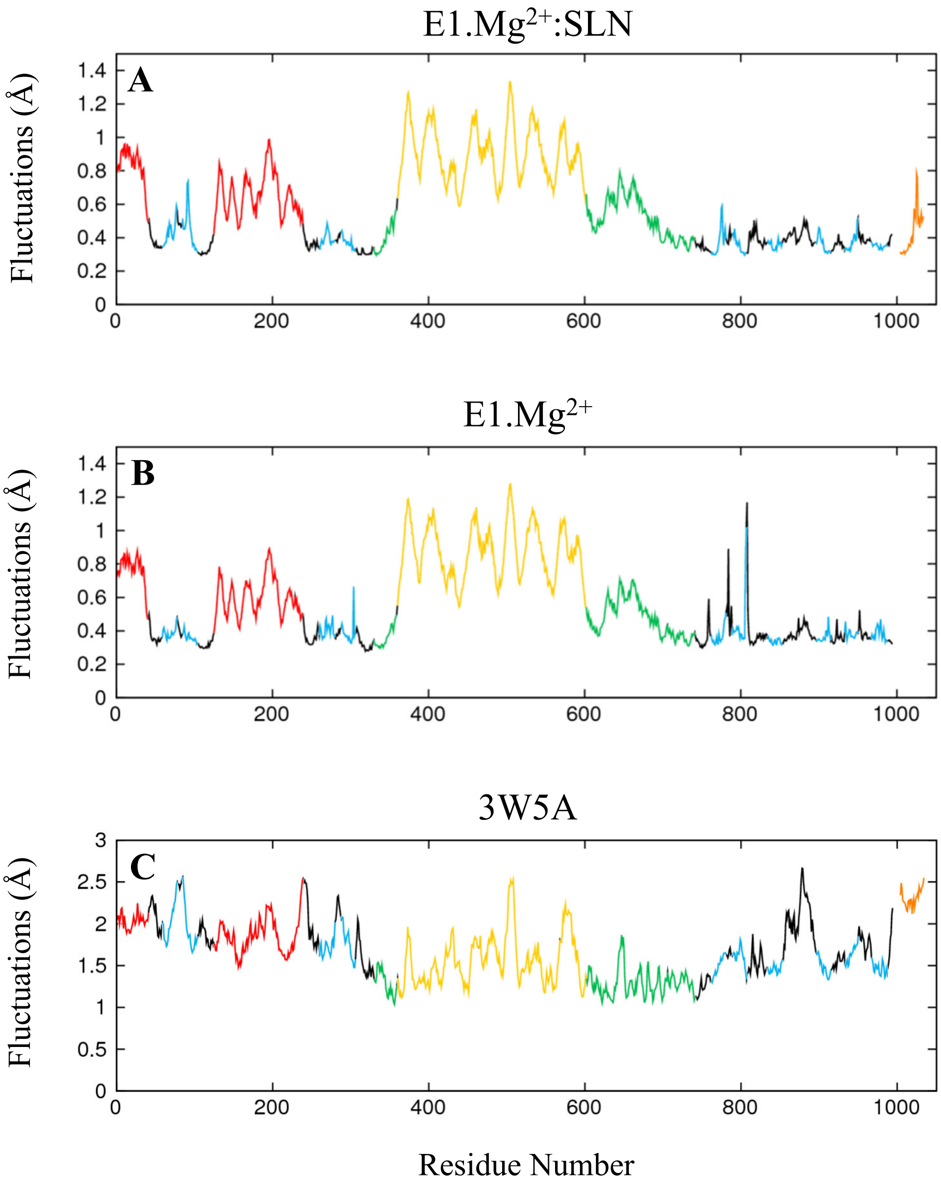

Figure S4. Fluctuations of the C_α atoms. The fluctuations are calculated from the 200 non-trivial modes of E1.Mg^{2+}:SLN (A) and E1.Mg^{2+} (B). (C) The fluctuations are calculated from the B-factors of the PDB structure 3W5A, chains B (SERCA1a) and C (SLN). The same color code as in Figure 1A in the main text is used to delimit the domains, and the fluctuations of SLN are in orange.

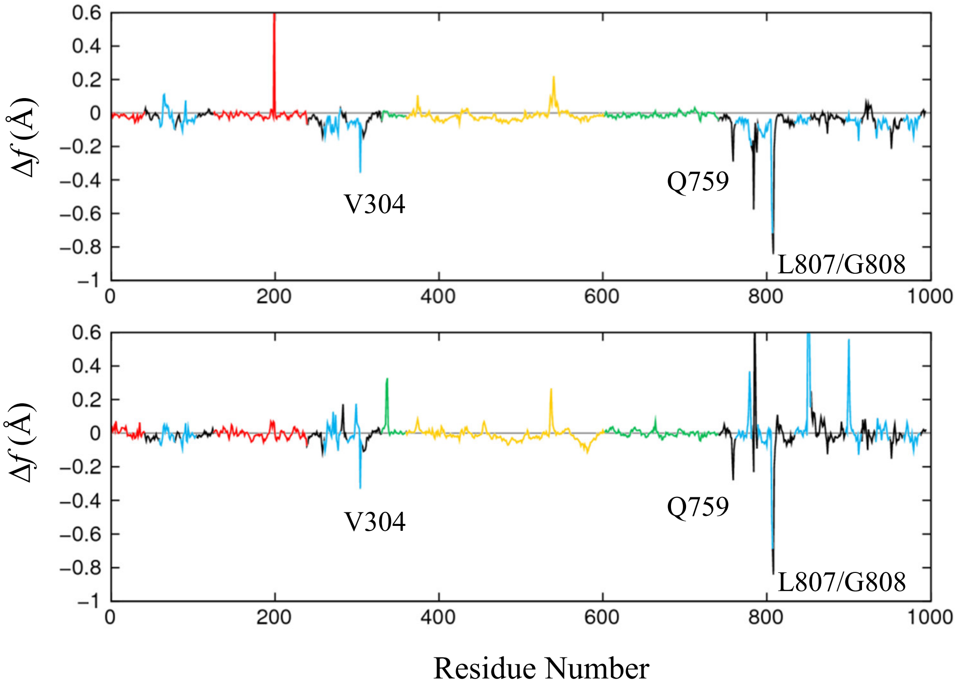

Figure S5. Difference of fluctuations of the C_α atoms. Δf, the difference of fluctuations of the C_α atoms calculated from the 200 modes. The fluctuations of E1.Mg^{2+} were subtracted from those of the two E1.Mg^{2+}:SLN structures that were not explicitly presented in the manuscript. The same color code as in Figure 1A in the main text is used to delimit the domains.

Table S3: Distances in the metal binding sites. Columns 1- 3: Distances between the metal (either Mg^{2+} or Ca^{2+}) and the closest oxygen atom of three important chelating residues. Column 4: distances between atom $N_{\delta 2}$ of N768 and the closest oxygen $O_{\delta *}$ from the sidechain of D800. In the title column, in the first four rows are given the PDB IDs of the structures with their corresponding states in parenthesis, and in the last two rows are presented the energy-minimized structures starting from 3W5A – chain B, in the presence and absence of SLN.

Distance (Å) / Structure (state)	Metal – D800	Metal – E771	Metal – N768	N768-$N_{\delta 2}$ – D800-$O_{\delta *}$
**3W5A– chain B (E1.Mg^{2+}:SLN)**	2.87	2.72	2.52	5.65
**4H1W (E1.2Mg^{2+}:SLN)**	2.16	5.46	4.38	7.36
**3W5B (E1.Mg^{2+})**	3.69	2.50	2.31	4.53
**1VFP – chain A (E1.2Ca^{2+})**	2.26 – 2.33	2.41	2.53	3.08
**E1.Mg^{2+}:SLN**	4.22	1.99	2.12	4.83
**E1.Mg^{2+}**	3.92	1.99	2.48	3.40

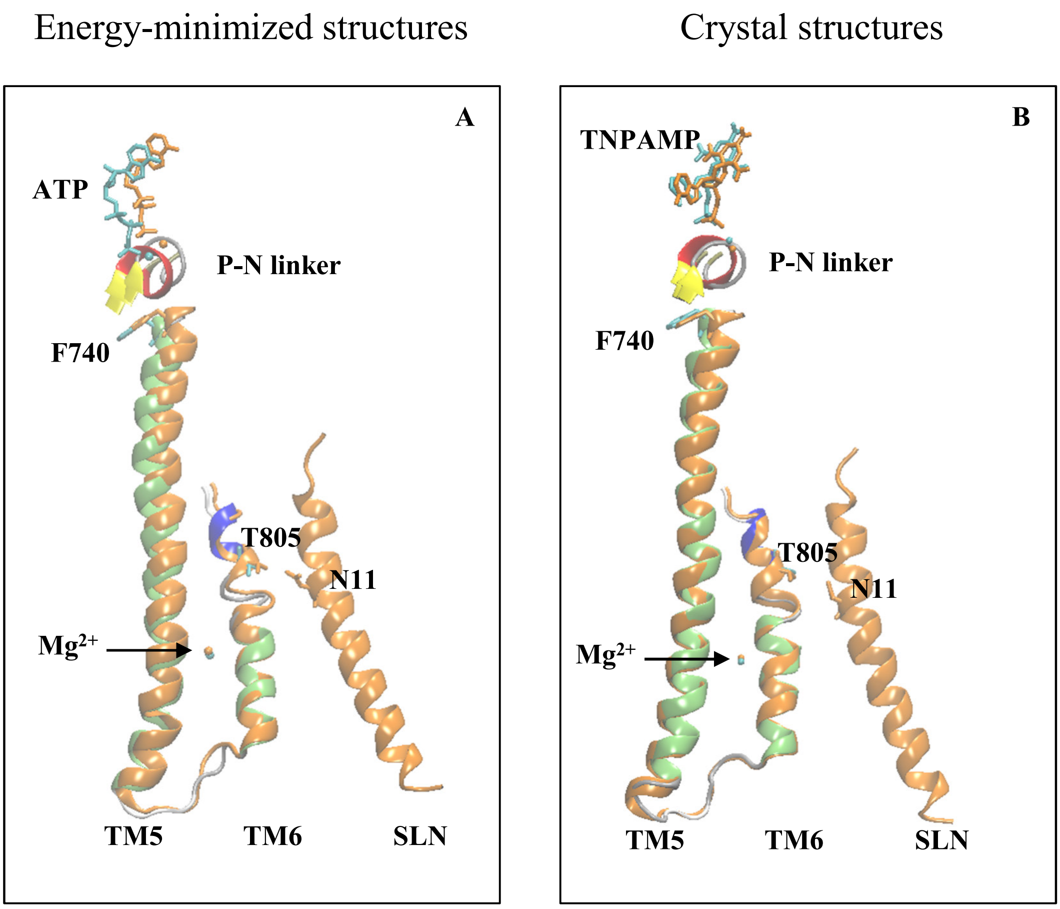

Figure S6. Comparison of the difference between E1.Mg^{2+} and E1.Mg^{2+}:SLN for the energy-minimized structures (A) and the crystal structures (B). The PDB structures are 3W5A and 3W5B and the energy-minimized structures are both derived from 3W5A. In each case, the backbone of the α-helix part of TM6 (residues 790 to 799) of the two structures were superimposed. The color code is as follows: all the structure elements in the presence of SLN are in orange, except for the P-N linker which is in gray. In the absence of SLN, α-helices are in light green, the TM6 3-10 helix in dark blue, the P-N linker π-helix in red, the end of the second β-strand in yellow, the unstructured regions in gray. ATP, TNPAMP, F740, T805 and Mg^{2+} are in cyan sticks.

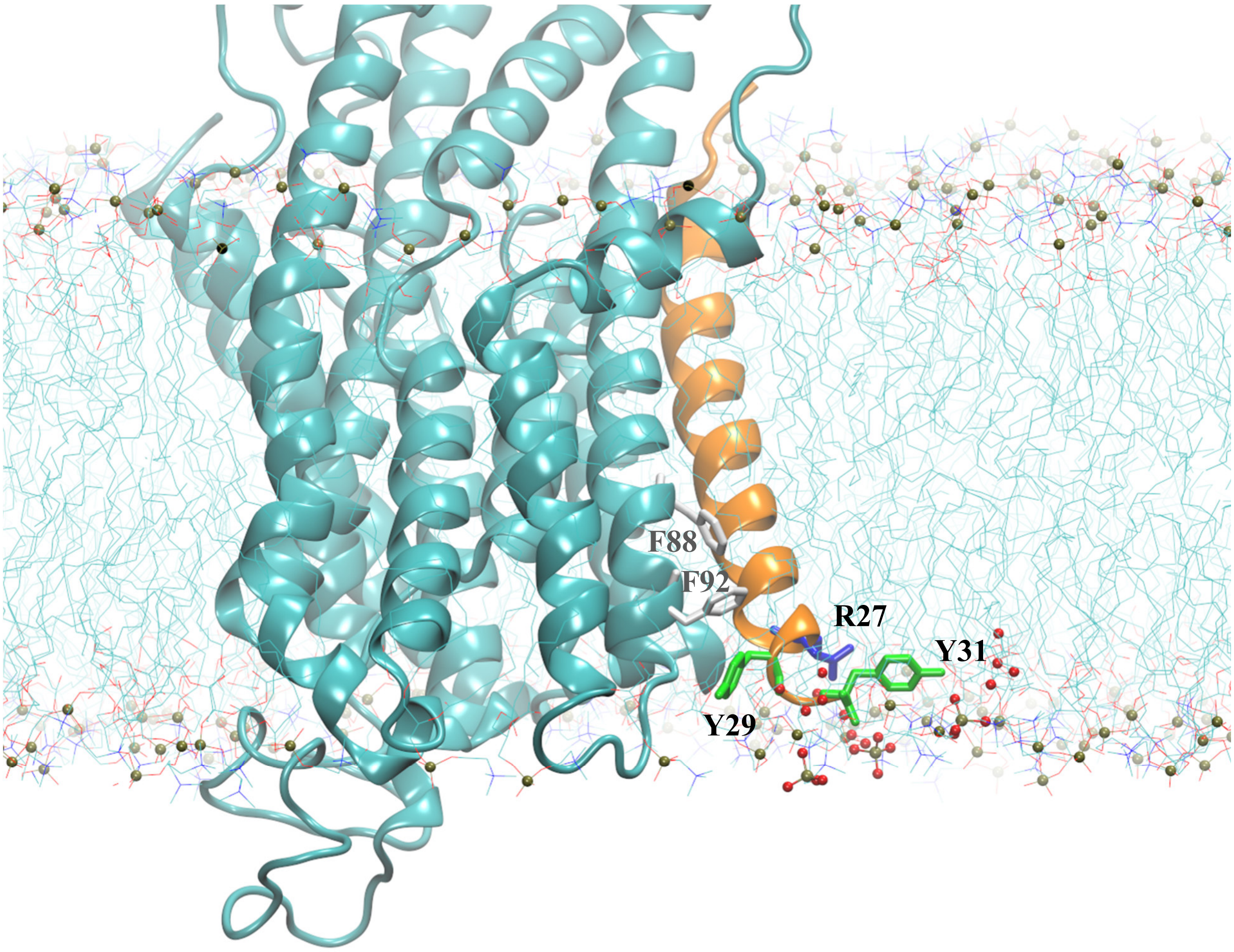

Figure S7. Zoom on the C-terminal tail of SLN. The energy-minimized structure E1.Mg^{2+}:SLN is drawn in the presence of the membrane, where SERCA1a is in cyan cartoon and SLN in orange. Three residues of SLN, R27, Y29, and Y31, and two residues of SERCA1a, F88 and F92, are drawn as sticks, and colored according to the residue type: blue for basic, green for polar, and white for hydrophobic. The membrane is in cyan lines with phosphorus atoms in tan spheres. The oxygen atoms of the lipids that interact with the two essential residues R27 and Y31 are shown as red spheres.

REFERENCES

[1]Toyoshima, C., and T. Mizutani. 2004. Crystal structure of the calcium pump with a bound ATP analogue. Nature. 430:529–535.

[2]Toyoshima, C., S.-I. Yonekura, J. Tsueda, and S. Iwasawa. 2011. Trinitrophenyl derivatives bind differently from parent adenine nucleotides to Ca^{2+}-ATPase in the absence of Ca^{2+}. Proc. Natl. Acad. Sci. USA. 108:1833–1838.

[3]Sørensen, T.L.-M., J.V. Møller, and P. Nissen. 2004. Phosphoryl transfer and calcium ion occlusion in the calcium pump. Science. 304:1672–1675.

[4]Sacchetto, R., I. Bertipaglia, S. Giannetti, L. Cendron, F. Mascarello, E. Damiani, E. Carafoli, and G. Zanotti. 2012. Crystal structure of sarcoplasmic reticulum Ca^{2+}-ATPase (SERCA) from bovine muscle. J. Struct. Biol. 178:38–44.

[5]Toyoshima, C., H. Nomura, and T. Tsuda. 2004. Lumenal gating mechanism revealed in calcium pump crystal structures with phosphate analogues. Nature. 432:361–368.

[6]Clausen, J.D., M. Bublitz, B. Arnou, C. Olesen, J.P. Andersen, J.V. Møller, and P. Nissen. 2016. Crystal Structure of the Vanadate-Inhibited Ca^{2+}-ATPase. Struct. Lond. Engl. 1993. 24: 617–623.

[7]Olesen, C., M. Picard, A.-M.L. Winther, C. Gyrup, J.P. Morth, C. Oxvig, J.V. Møller, and P. Nissen. 2007. The structural basis of calcium transport by the calcium pump. Nature. 450:1036–1042.

[8]Toyoshima, C., S. Iwasawa, H. Ogawa, A. Hirata, J. Tsueda, and G. Inesi. 2013. Crystal structures of the calcium pump and sarcolipin in the Mg^{2+}-bound E1 state. Nature. 495: 260–264.

[9]Toyoshima, C., Norimatsu, Y., Tsueda, J., 2007. Crystal structure of the calcium pump with AMPPCP in the absence of calcium. https://doi.org/10.2210/pdb2dqs/pdb.

[10]Akin, B.L., T.D. Hurley, Z. Chen, and L.R. Jones. 2013. The structural basis for phospholamban inhibition of the calcium pump in sarcoplasmic reticulum. J. Biol. Chem. 288:30181–30191.

[11]Winther, A.-M.L., M. Bublitz, J.L. Karlsen, J.V. Møller, J.B. Hansen, P. Nissen, and M.J. Buch-Pedersen. 2013. The sarcolipin-bound calcium pump stabilizes calcium sites exposed to the cytoplasm. Nature. 495:265–269.
